# Topoisomerase I Plays a Critical Role in Suppressing Genome Instability at a Highly Transcribed G-Quadruplex-Forming Sequence

**DOI:** 10.1371/journal.pgen.1004839

**Published:** 2014-12-04

**Authors:** Puja Yadav, Victoria Harcy, Juan Lucas Argueso, Margaret Dominska, Sue Jinks-Robertson, Nayun Kim

**Affiliations:** 1 Department of Microbiology and Molecular Genetics, University of Texas Health Science Center at Houston, Houston, Texas, United States of America; 2 Department of Environmental and Radiological Health Sciences, Colorado State University, Fort Collins, Colorado, United States of America; 3 Department of Molecular Genetics and Microbiology, Duke University Medical Center, Durham, North Carolina, United States of America; Stanford University, United States of America

## Abstract

G-quadruplex or G4 DNA is a non-B secondary DNA structure that comprises a stacked array of guanine-quartets. Cellular processes such as transcription and replication can be hindered by unresolved DNA secondary structures potentially endangering genome maintenance. As G4-forming sequences are highly frequent throughout eukaryotic genomes, it is important to define what factors contribute to a G4 motif becoming a hotspot of genome instability. Using a genetic assay in *Saccharomyces cerevisiae*, we previously demonstrated that a potential G4-forming sequence derived from a guanine-run containing immunoglobulin switch Mu (Sμ) region becomes highly unstable when actively transcribed. Here we describe assays designed to survey spontaneous genome rearrangements initiated at the Sμ sequence in the context of large genomic areas. We demonstrate that, in the absence of Top1, a G4 DNA-forming sequence becomes a strong hotspot of gross chromosomal rearrangements and loss of heterozygosity associated with mitotic recombination within the ∼20 kb or ∼100 kb regions of yeast chromosome V or III, respectively. Transcription confers a critical strand bias since genome rearrangements at the G4-forming Sμ are elevated only when the guanine-runs are located on the non-transcribed strand. The direction of replication and transcription, when in a head-on orientation, further contribute to the elevated genome instability at a potential G4 DNA-forming sequence. The implications of our identification of Top1 as a critical factor in suppression of instability associated with potential G4 DNA-forming sequences are discussed.

## Author Summary

Genome instability is not evenly distributed, but rather is highly elevated at certain genomic loci containing DNA sequences that can fold into non-canonical secondary structures. The four-stranded G-quadruplex or G4 DNA is one such DNA structure capable of instigating transcription and/or replication obstruction and subsequent genome instability. In this study, we used a reporter system to quantitatively measure the level of genome instability occurring at a G4 DNA motif integrated into the yeast genome. We showed that the disruption of Topoisomerase I function significantly elevated various types of genome instability at the highly transcribed G4 motif generating loss of heterozygosity and copy number alterations (deletions and duplications), both of which are frequently observed in cancer genomes.

## Introduction

In addition to the canonical Watson-Crick helical duplex (B-DNA), genomic DNA, especially repetitive sequences, can assume other types of structures such hairpins, Z-DNA, triplex DNA (H-DNA) or tetrahelical DNA structures [1]–[6]. Impediments to normal DNA metabolic processes including transcription and replication imposed by such secondary DNA structures explain the correlation between repetitive sequence elements and elevated genome instability. Genomic instability at purine-rich GAA•TTC repeats and CAG•CTG repeats, which can fold into three-stranded H-DNA [7] and a slipped hairpin structure [8], forms the molecular basis of multiple neurodegenerative diseases, such as Freidreich's Ataxia and Huntington's disease, respectively.

G-quadruplex or G4 DNA is another non-B secondary DNA structure that potentially interferes with normal DNA transactions [5], [9]–[11]. G4 DNA contains a stacked array of multiple G-quartets, which are comprised of four guanines interacting in a planar configuration [5], [10]. G4 DNA can be readily formed in solution by oligonucleotides containing multiple runs of guanines and by actively transcribed plasmid DNA [12], [13]. G4 DNA-forming sequences or G4 motifs are present in the genomes of diverse organisms and conserved throughout evolution; they number >375,000 in the human genome and >1,400 in the *Saccharomyces cerevisiae* nuclear genome [14]–[17]. The distribution of G4 motifs is highly concentrated at telomeres, rDNA loci, immunoglobulin heavy-chain switch regions, and G-rich minisatellites and significantly correlates with nucleosome-free regions and transcription start sites (TSSs) [10], [11], [18]. In oncogenes, G4 motifs are mostly enriched in the regions flanking TSSs, which suggests that G4 DNA may be involved in transcriptional regulation [15], [17].

G4 DNA becomes a structural barrier to transcription and replication *in vitro* indicating that it might play a significant role in genome instability [19]–[21]. In the absence of Pif1, a potent G4 DNA unwinding helicase, replication forks slow down near G4 motifs present in the yeast genome strengthening the argument that an unresolved G4 structure can lead to increased genome instability [22]. G4 motifs are frequently found at unstable genomic loci including proto-oncogenes and sites of frequent translocation breakpoints [23] and at preferred mitotic and meiotic DNA break sites [17]. In some human cancers, G4 motifs have been identified at frequent breakpoints involved in chromosomal translocations including the major breakpoint region in the proto-oncogene BCL2 [24]. Chromosomal translocations involving G-rich immunoglobulin switch regions have long been observed in various cancer cell lines [25]. The identification *in silico* of potential G4 DNA-forming sequences at sites of genome instability, however, has not yet been fully verified by *in vivo* demonstration of biological relevance of G4 DNA structure.

Seminal advances in understanding the genome instability induced by the repetitive, DNA secondary structure-forming sequences have been made using yeast and bacterial model systems [26]–[28]. A large tract of GAA•TTC repeats in yeast, for example, acted as a hotspot of gross chromosomal rearrangements (GCRs) and interstitial deletions [26], [27]. In bacteria, CTG•CAG repeats from the Myotonic Dystrophy gene induced large deletions when the repeats were highly transcribed [29]. When a guanine-run containing a human subtelomeric minisatellite was integrated into the yeast genome, it significantly elevated GCRs [30] and resulted in frequent repeat expansion and contraction [31]. The guanine-rich yeast telomere repeats, when placed within an intron of an interstitially located gene, cause various types of chromosomal rearrangements including deletions and inversions [32]. Accumulating evidence pointing to G4 motifs as genome instability hotspots underscores the importance of defining endogenous and exogenous factors that influence the integrity of genomic loci containing these motifs.

Active transcription, when oriented in the direction to place the guanine-runs in the transiently single stranded non-transcribed strand (NTS), was shown to stimulate formation of G4 DNA structure both *in vitro* and in bacterial cells [13]. To determine the effect of G4 DNA on yeast genome stability, we previously constructed a genomic reporter assay where a potential G4 DNA-forming sequence was highly transcribed to promote secondary DNA structure formation. By normalizing the extent of genome instability occurring at this reporter construct to that occurring at the exact same sequence transcribed in inverse orientation (that is, with the G4 motifs on the transcribed strand), we were able to apply a stringent control for the correlation between elevated genome instability and G4 DNA. Using this approach, we found that gene conversion recombination was significantly elevated by highly transcribing guanine-run containing sequence in a strictly strand-specific manner [33].

In the current report, we demonstrate that active transcription transforms a guanine-run containing sequence into a strong hotspot for gross chromosomal rearrangements and loss-of-heterozygosity (LOH). Our data also show that the direction of replication can significantly alter the level of instability at a potential G4 DNA-forming sequence suggesting that genomic context in terms of both transcription and replication is important when considering G4 motifs as potential genome instability hotspots. Finally, we identify a critical role of Topoisomerase I (Top1) in suppressing various types of genome rearrangements associated with co-transcriptionally formed G4 DNA.

## Results

### A genomic assay to measure gross chromosomal rearrangements

**Figure 1:**
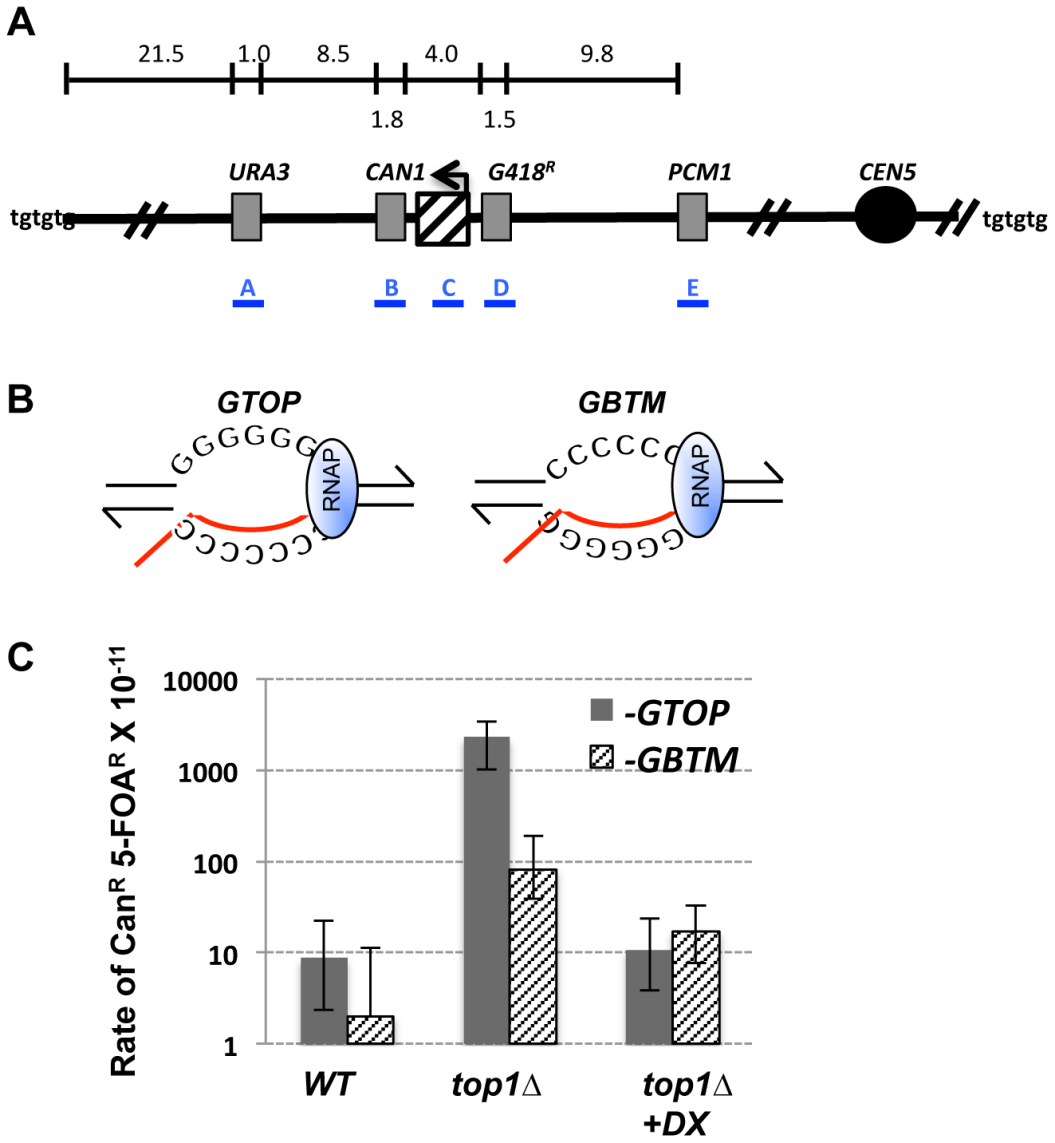
Gross Chromosomal Rearrangement (GCR) assay. A. A schematic representation of the GCR assay. The location of *pTET-lys2-GTOP* or *–GBTM* cassette is indicated by the hashed box with the arrow above indicating the direction of transcription. The distances (in kb) are approximate and not to scale. Telomeres are represented as “tgtgtg”. Blue bars with labels A, B, C, D, and E represents the approximate location of the PCR products used for characterization of GCR products (see Table 1). B. The transcription orientations of the Sμ-containing cassettes. Guanine-runs are on the non-transcribed strand in single stranded state in *pTET-lys2-GTOP* cassette and on the transcribed strand annealed to the nascent RNA (red line) in the *pTET-lys2*–*GBTM* cassette. RNA polymerase holoenzyme is indicated by the blue oval. C. The rates of GCRs occurring at CHR5 containing the *pTET-lys2-GTOP* (gray bar) or *–GBTM* (hashed bar) cassette. +DX; Doxycycline was added to the final concentration of 2 µg/ml to repress the transcription from *pTET*. The relative RNA levels under high and low transcription conditions are shown in Table S1. *; the rates determined by the *p_0_* method. **; the rates determined by the method of median. 95% confidence intervals are indicated by the error bars.

We previously showed that gene conversion resulting from ectopic recombination is increased due to co-transcriptionally formed G4 DNA. In order to determine whether co-transcriptionally formed G4 DNA can also elevate gross chromosomal rearrangements (GCRs), we modified the GCR reporter system previously described by Chen and Kolodner [34], [35]. In this reporter system, the *URA3* gene was integrated into the left arm of chromosome V (CHR5) replacing the *HXT13* gene located ∼8.5 kb centromere-distal to the *CAN1* gene (Fig. 1A). The loss of functional *CAN1* or *URA3* results in resistance to the drug canavanine (Can) or 5-Fluoroorotic acid (5-FOA), respectively. Because the first essential gene on the left arm of CHR5, *PCM1*, is located ∼60 kb from the telomere, the region containing *CAN1* and *URA3* genes can be lost without affecting viability of haploids. The hypothetical rate of double drug resistance (Can^R^/5-FOA^R^) occurring *via* independent mutations in *CAN1* and *URA3* is approximately 10^−12^, which is significantly lower than the observed rate of deletion of the left arm of CHR5 (10^−11^–10^−10^ in a wild-type background) [35]. Therefore, by selecting for colonies resistant to both Can and 5-FOA, GCR events resulting in simultaneous loss of *CAN1* and *URA3* genes are detected.

We modified the GCR assay by integrating the *pTET-lys2-GTOP* or -*GBTM* cassette immediately centromere-proximal to *CAN1* (Fig. 1A). As previously described [36], the *pTET-LYS2* cassette contains the *LYS2* gene transcribed from the heterologous tetracycline-repressible promoter (*pTET*) linked to a marker gene conferring G418 resistance (G418^R^). Within the *LYS2* ORF, at ∼390 bp from the start codon, a 760 bp fragment from the mouse immunoglobulin (Ig) switch Mu (Sμ) region was inserted to generate *pTET-lys2-GTOP* or -*GBTM* cassettes [33]. Switch regions, which are required for Ig heavy chain class-switch recombination (CSR), are comprised of multiple, degenerate guanine-rich repeats of several kb in length [37]. The Sμ sequence is a model G4 motif and was previously demonstrated to form G4 DNA structures both *in vivo* and *in vitro* when highly transcribed [13]. The sequence of the Sμ fragment incorporated into the *LYS2* ORF (containing ∼17 of (GAGCT)_n_ GGGGT repeats) is shown in Figure S1. This fragment was inserted either in the physiological (-*GTOP*) or in the inverted orientation (*-GBTM*), placing the guanine runs on the non-transcribed (NTS) or the transcribed strand (TS), respectively (Fig. 1B) [33].

### GCR is elevated when Top1 is disrupted under high-transcription conditions

Topoisomerase 1 (Top1) is a highly conserved enzyme that relieves positive or negative torsional stress associated with transcription and replication [38], [39]. Top1 functions by covalently attaching to the 3′ end of nicked DNA, which is quickly re-ligated after swiveling of the DNA strands to remove supercoiling. Although not essential for viability in yeast, replication slows down and sometimes stalls in the absence of Top1, especially at highly transcribed regions [40]. We previously reported that disruption of Top1 leads to an increase in Sμ-induced gene conversion events [33].

In order to determine whether Top1 also plays a role in preventing GCRs initiating at G4 DNA, we deleted the *TOP1* gene from strains containing *pTET-lys2-GTOP* and *–GBTM* constructs. In wild-type (*WT*) backgrounds under high-transcription conditions, the rates of Can^R^/5-FOA^R^ events were 0.88×10^−10^ and 0.20×10^−10^ for *pTET-lys2-GTOP* and *–GBTM* constructs, respectively (Fig. 1C). These rates are not significantly different from each other and are comparable to GCR rates previously reported in the absence of an inserted *pTET-lys2* cassette [34], [35]. Upon *TOP1* deletion, the rate of GCR (Can^R^/5-FOA^R^) was significantly elevated for both the *pTET-lys2-GTOP* and *-GBTM* construct. Importantly, GCR occurred at a significantly higher (∼30-fold) rate for *pTET-lys2–GTOP*, where guanine-runs are present on the NTS, compared to the *pTET-lys2–GBTM* construct where guanine-runs are on the TS.

We tested whether active transcription of the guanine-run-containing sequence is required for the elevated GCRs by growing the *top1Δ* strains in medium containing doxycycline, an analog of tetracycline, which resulted in ∼60- to 200-fold reductions in the transcription rates (Table S1). Repression of transcription from the *pTET* promoter by doxycycline resulted in a >200-fold decrease in the rate of Can^R^/5-FOA^R^ for the strain associated with the *pTET-lys2-GTOP* construct (Fig. 1C). For the strain containing the *pTET-lys2-GBTM* construct, transcriptional repression also led to a significant decrease in the rate of Can^R^/5-FOA^R^.

### In the absence of Top1, GCR events mainly occur proximal to G4 motifs and are frequently resolved by *de novo* telomere addition

In order to determine whether the GCRs in *top1Δ* backgrounds initiate at the G4 DNA containing reporter construct, we carried out PCR analysis to map the GCR initiating breakpoints. For the strain containing the *pTET-lys2-GTOP* construct in the *top1Δ* background, 27 of 30 Can^R^/5-FOA^R^ isolates tested had lost the portion of *LYS2* gene with the Sμ fragment insertion but still retained the G418^R^ cassette located just centromere-proximal to this region (Fig. 1A and Table 1). Thus, 90% of the GCR events occurring in this strain initiated within the ∼4 kb region comprised of the *pTET-lys2-GTOP* cassette between G418^R^ cassette and *CAN1*. This is proportionally greater than the GCR initiating in the same region for the *pTET-lys2-GBTM* construct in *top1Δ* strain (9 out 23; P<0.0005 by chi square analysis).

**Table 1:** PCR analysis to map GCR breakpoints.

*PCR Fragments*					*G4 Construct*	
*A (URA3)*	*B (CAN1)*	*C (lys2* * *)*	*D (G418^R^)*	*E (PCM1)*	*pTET-lys2-GTOP (N = 30)*	*pTET-lys2-GBTM (N = 23)*
+	+	+	+	+	0	5
−	−	+	+	+	3	5
−	−	−	+	+	27	9
−	−	−	−	+	0	4

*PCR primers were designed to anneal to *LYS2* sequence upstream and downstream of the embedded Sμ fragment (*GTOP* or *GBTM*). See Fig. 1A for the approximate location of the PCR fragments analyzed. + indicates the presence of PCR product and − indicates the absence.

**Figure 2:**
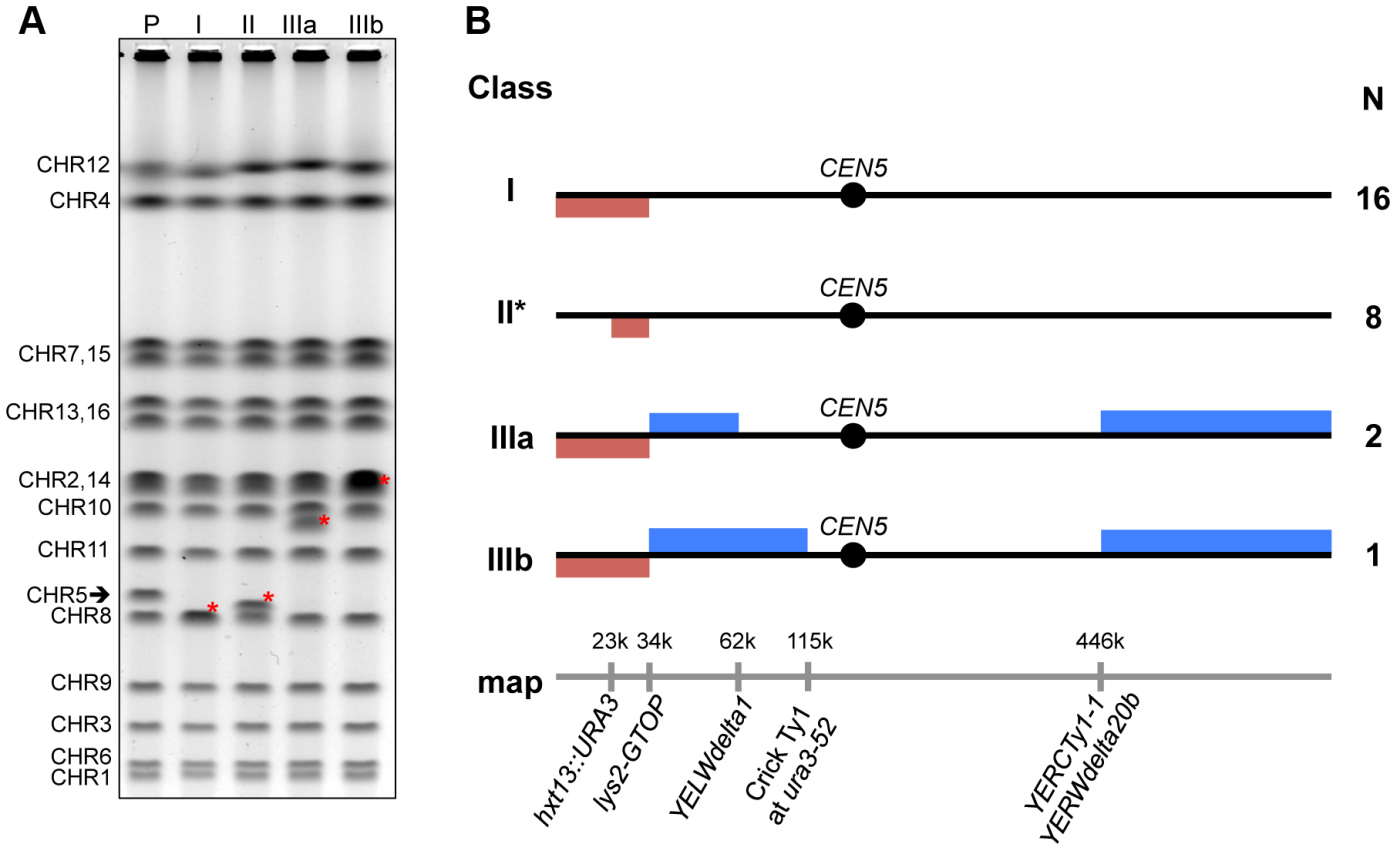
Classes of GCRs associated with the highly transcribed G4 DNA motif. A. PFGE analysis of different classes of GCR events. The full-length chromosomes of representative 5-FOA^R^ Can^R^ clone derived from the *top1Δ* strain containing the *pTET-lys2-GTOP* cassette are separated out by size. Classes I, II, IIIa and IIIb are described in the text. The chromosomes in the parental strain (P) are indicated to the left and the arrow indicates the normal (unrearranged) CHR5. The locations of novel chromosomal bands in each sample are indicated by red asterisks. B. Summary of the array-CGH analyses. The red and blue bars indicate the loss and the gain of the copy number of the corresponding regions of CHR5, respectively. The coordinates are approximate and correspond to the S288c reference genome sequence according to Saccharomyces Genome Database. The relevant genomic features are indicated at the bottom of the figure. The locations of relevant solo LTRs and full length Ty retrotransposons are indicated. At coordinate ∼115,000, the *ura3-52* allele with a Crick-oriented insertion of a Ty1 element is present in this strain background. The quantitative analysis of the PFGE in Fig. 2A and the detailed CHR5 gene dosage plots for the GCR classes are shown in Fig. S2. The detailed breakpoint nucleotide sequence of the segmental deletion in Class II events (*) is shown in Figure S3.

To further characterize the chromosome breakpoints in GCR events associated with the *pTET-lys2-GTOP* cassette, we carried out PCR with a degenerate primer annealing to generic yeast telomere sequence (CA16; 5′ CACCACACCCACACAC 3′) and a primer annealing to the 5′ untranslated region of the *LYS2* gene. Out of 27 samples where the disruption of the *pTET-lys2-GTOP* cassette was confirmed by PCR mapping, telomere-anchored PCR products of 700 to 1900 bp were obtained for 16 samples. Subsequent sequencing of these fragments showed that, in 15 Can^R^/5-FOA^R^ clones, *de novo* telomere additions occurred at various locations within the G4-forming Sμ fragment (Class I events in Fig. 2 and Fig. S1). Due to the high G/C content and repetitiveness of the Sμ sequence, sequencing analysis failed to identify the site of telomere addition in one of the PCR fragments. In concurrence with preferential telomere addition sites previously identified [41], the junctions of *de novo* telomere addition were located at GT dinucleotides and frequently at 5 to 6 nt clusters of GT-rich sequence. Additionally, mutations, deletions/insertions, and duplications within the Sμ fragment were detected in seven of the 15 Can^R^/5-FOA^R^ clones with telomere additions.

### The actively transcribed G4 motif induces complex genome rearrangements involving deletions and inverted duplications

We further characterized the genome rearrangements associated with G4 DNA using pulse field gel electrophoresis (PFGE) and microarray-based comparative genome hybridization (array-CGH). PFGE showed that, in the 16 samples where *de novo* telomere addition at Sμ was confirmed by sequencing (Class I), CHR5 was reduced in size by ∼35 kb and co-migrated with CHR8 (Fig. 2A and Fig. S2A). The loss of CHR5 sequences from the left telomere to the integration site of *pTET-lys2* cassette (at 34,000 NT) was confirmed by array-CGH analysis (Fig. 2B and Fig. S2A).

In another 8 samples (Class II) where CHR5 appeared smaller by only ∼15 kb (Fig. 2A and Fig. S2B), array-CGH identified segmental deletions between the original location of *HXT13* (at 23,000 NT) and the *pTET-lys2* cassette. PCR and sequencing analysis showed that this recurrent deletion was mediated by a pair of 21 bp direct repeats introduced into the two respective sites of CHR5 as parts of plasmid constructs used for integration of the *URA3Kl* marker at *HXT13* locus (pUG72; Euroscarf) and *pTET* promoter (pCM225; Euroscarf) (Fig. S3).

Finally, in class III events, deletion of CHR5 sequences from the left telomere to the *pTET-lys2* integration site occurred in combination with duplications of the immediate proximal sequences on the left arm, and duplication of a terminal segment on the right arm (Fig. 2B and Fig. S2C–D). The two clones from class IIIa showed duplications from *pTET-lys2* to the Watson-oriented *YELWdelta1* dispersed long terminal repeat element (LTR), and the one example of class IIIb showed a duplication extending further to the full length Crick-oriented Ty1 element insertion at the *URA3* locus (*ura3-52* allele). All three class III clones had duplications of a segment of the right arm extending from position ∼446,000 NT (containing the full length Crick-oriented *YERCTy1-1* element and the Watson-oriented *YERWdelta20b* LTR) all the way to the right telomere. These clones displayed longer versions of CHR5 of ∼700 kb (migrating just below CHR10) and ∼770 kb (co-migrating with CHR2 and CHR14) for class IIIa and class IIIb, respectively (Fig. 2A and Fig. S2C–D).

The class III chromosome sizes were consistent with the deletions and duplications detected by array-CGH and suggested a complex mechanism of formation. Similar array-CGH patterns were observed recently in the analyses of GCR events in yeast CHR5 as well as in humans [42], [43]. These studies described breakpoint structures consistent with an intra-strand fold-back mechanism in which a resected free 3′ end folds back on itself, re-anneals to a microhomology region and primes break-induced DNA replication (BIR). By plasmid-rescuing this regions subcloning and sequence analysis (See Materials and Methods), we confirmed that the duplicated regions proximal to the Sμ breakpoints in class IIIb clone A7 and class IIIa clone A8 were comprised of inverted duplications separated by single copy regions corresponding to the original ssDNA loops, as predicted by the fold-back mechanism (Figs. S4A and S4B). We were not successful in rescuing the duplicated regions from the Class IIIa clone A16. The recovered rearrangement structures observed in the A7 and A8 clones can be explained by two different models (Fig. S4C). In the first scenario, as the BIR event initiated by the intra-strand fold-back reached the Ty/LTR sequences on the left arm, it collapsed, re-annealed at the Ty/LTR sequences on the right arm (template switching), and continued on to reach the right telomere to produce a stable monocentric chromosome. The second possibility is that BIR continued all the way to the right telomere forming an unstable dicentric chromosome, which was then stabilized by a secondary homologous recombination event between Ty/LTR repeats leading to loss of one of the centromeres. Although our data does not allow us to distinguish between these two possibilities, we favor the template switching model (Model 1 in Fig. S4C) since BIR is generally thought to be impeded by centromeric structures, and BIR template switching has been shown to be frequent in yeast [44], [45].

### A diploid system to examine the effect of G4 motifs on mitotic recombination

**Figure 3:**
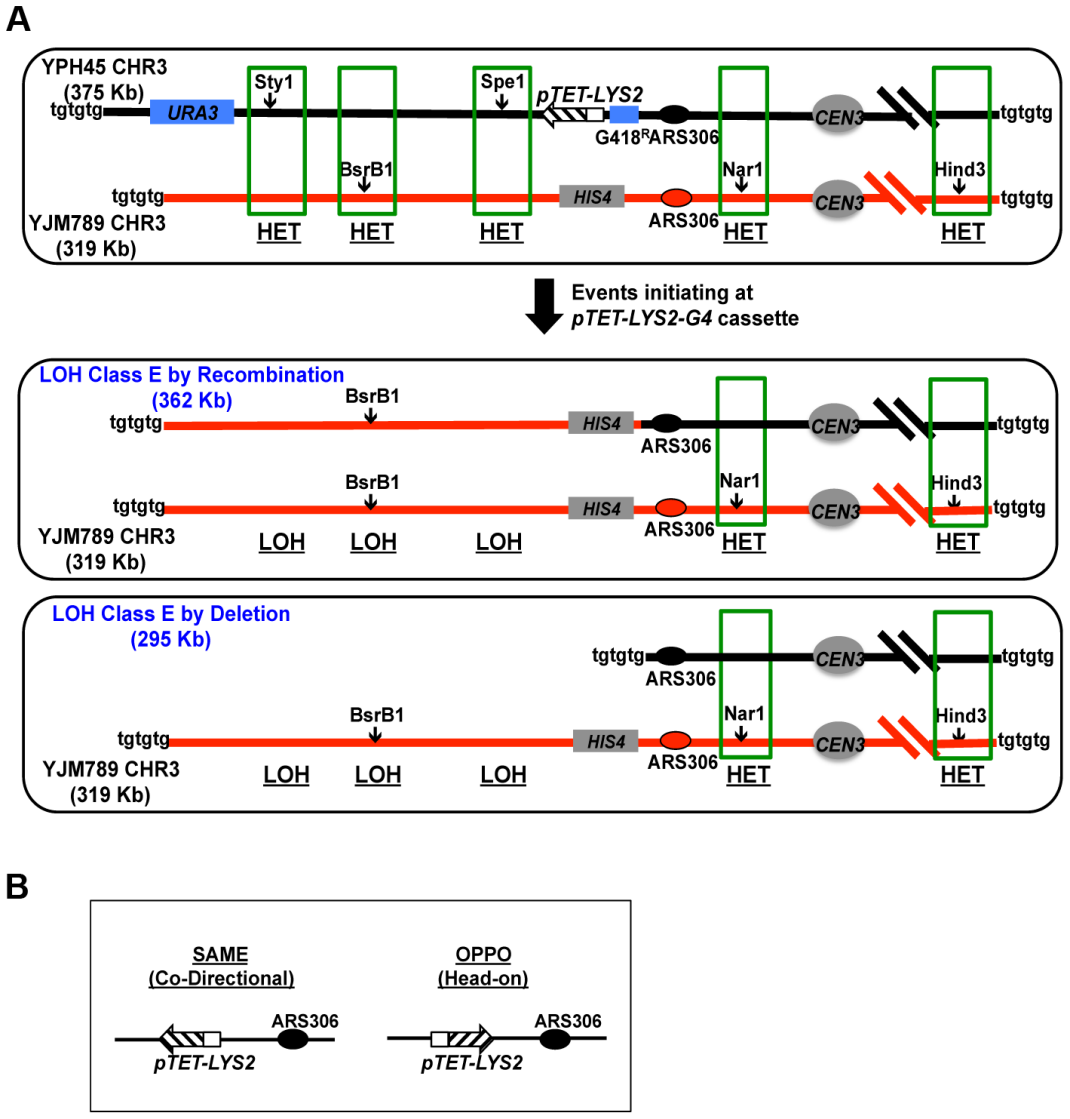
Loss of Heterozygosity (LOH) assay. A. A schematic representation of the LOH assay in heterozygous diploid yeasts (YPH45×YJM789). YPH45-CHR3 and YJM789-CHR3 are represented by black and red lines, respectively. Location of the *pTET-lys2-GTOP* or *–GBTM* cassette on the left arm of CHR3 is indicated by the hashed box. Green boxes indicate the approximate locations of the heterozygous SNP markers listed in Table S1. Telomeres are represented as “tgtgtg”. Hemizygous *URA3* and *G418^R^* markers present only on the YPH45-CHR3 homolog are indicated by blue boxes. The top panel and the bottom two panels represent the parental 5-FOA^S^ diploid and LOH Class E events as described in the text and in Table 2, respectively. LOH Class E events initiating at the *pTET-lys2-GTOP* or *–GBTM* cassette either by recombination or deletion will result in the same RFLP-SNP pattern but in the difference in size alterations of the YPH45 CHR3. B. The orientation of *pTET-lys2-GTOP* or *–GBTM* cassette insertion relative to *ARS306* in SAME or OPPO configuration.

Spontaneous DNA breaks in diploid cells are frequently repaired by allelic mitotic recombination using as template either a sister chromatid or a homologous chromosome. We designed an assay that can measure G4 DNA-induced mitotic recombination between chromosome III (CHR3) homologs in diploids (Fig. 3A). First, we integrated the *URA3* gene near the telomere of the left arm of CHR3 in a haploid strain derived from YPH45 (a S288c derivative). On the same arm of CHR3, about 44 kb centromere-proximal to the *URA3* integration site, *pTET-lys2-GTOP* or -*GBTM* was integrated replacing *HIS4*. As described above for the CHR5 GCR assay, the *pTET-lys2-GTOP* and -*GBTM* cassettes contained the 760-bp fragment of Sμ sequence and were adjacent to an aminoglycoside phosphotransferase gene conferring resistance to the drug G418 (G418^R^). Because the direction of replication fork movement relative to the direction of transcription can affect recombination at highly transcribed regions [46], [47], each cassette was integrated in two orientations relative to the nearby replication origin *ARS306*. This yielded constructs in which the transcription and replication forks are co-directional (SAME) or in head-on orientation (OPPO) (Fig. 3B). The direction of replication fork movement through this region of CHR3 was previously confirmed by 2D-gel analysis [36].

Heterozygous diploids were generated by mating the YPH45-derived haploid strains described above to a haploid strain derived from YJM789, a clinically isolated strain with ∼0.5% sequence divergence relative to the S288c reference strain [48]. Because the YJM789-derived strain is Ura^−^, loss or mutation of the *URA3* gene on the CHR3 from the YPH45 parent will result in resistance to 5-FOA in the heterozygous diploid cells. The types of genome rearrangements that can lead to loss of *URA3* include (a) complete loss of YPH45 CHR3, (b) partial loss of the left arm of YPH45 CHR3, (c) Break Induced Replication (BIR) or reciprocal crossover (RCO) initiating between *URA3* and *CEN3* or (d) translocation/BIR events involving a heterologous chromosome. In RCO, the distal ends of the two CHR3s are exchanged without loss of genetic material. In BIR involving the homolog, the break in YPH45-CHR3 will be repaired through replication using YJM789-CHR3 from the break to telomere as template. In our assay, we cannot distinguish between these two mechanisms (Fig. S5). Because all of these events result in loss of heterozygosity (LOH) for the segment of CHR3 containing the *URA3* marker, we hereafter refer to this assay as the LOH assay.

In order to map the position of LOH in 5-FOA^R^ isolates, we devised a PCR-based restriction fragment length polymorphism (RFLP-SNP) assay described in Fig. S6. We defined the initiation of recombination point as between the last telomeric SNP site displaying LOH and the first centromeric SNP site displaying heterozygosity (Table 2). A 5-FOA^R^ isolate was defined as resulting from recombination initiating at or near the G4 repeats (*G418-pTET-lys2-GTOP or GBTM*), when it was homozygous for the centromere-distal *Spe*I site and heterozygous for the centromere-proximal *Nar*I site and the G418^S^ cassette was not present (LOH class E in Table 2 and Figure 3A). When mitotic recombination is initiated by DNA breaks near the *pTET-lys2-GTOP* or –*GBTM* cassette, resection must extend into the region of the YPH45 CHR3 with homology to YJM789 CHR3 and, therefore, remove the *pTET-lys2-GTOP* or –*GBTM* cassette along with the G418^R^ marker.

**Table 2:** Mapping of LOH events by RFLP-SNP assay.

		SNP Markers*					LOH Class
*URA3*	*Sty*I	*BsrB*I	*Spe*I	G418^R^	*Nar*I	*Hind*III	
N	YPH/YJM	YPH/YJM	YPH/YJM	Y	YPH/YJM	YPH/YJM	A
N	YJM	YPH/YJM	YPH/YJM	Y	YPH/YJM	YPH/YJM	B
N	YJM	YJM	YPH/YJM	Y	YPH/YJM	YPH/YJM	C
N	YJM	YJM	YJM	Y	YPH/YJM	YPH/YJM	D
**N**	**YJM**	**YJM**	**YJM**	**N**	**YPH/YJM**	**YPH/YJM**	**E**
N	YJM	YJM	YJM	N	YJM	YPH/YJM	F
N	YJM	YJM	YJM	N	YJM	YJM	G

*The location and sequences of the SNP markers in YPH45 or YJM789 strain backgrounds are listed in Table S2. For *URA3* and G418^R^, PCR and agarose gel electrophoresis is carried out to verify the presence or the absence of the corresponding sequences (N- PCR product absent; Y – PCR product present). For *Sty*I, *BsrB*I, *Spe*I, *Nar*I, and *Hind*III, PCR products are digested with the corresponding restriction enzyme and run on agarose gel to verify presence of YJM789-SNP or YPH45-SNP. For example, 700 nt region including the *Sty*I-SNP site is amplified and digested with *Sty*I. If both YJM789 and YPH45 are present, there will be three bands – 700 nt uncut band from YJM789 and 450 nt and 250 nt bands resulting from enzyme digest of the PCR product amplified from YPH45 sequence. If the region of YPH45 containing the *Sty*I-SNP site is lost due to recombination, PCR/restriction digest will only result in the 700 nt uncut band from YJM789 sequence. YJM – YJM789 sequence present; YPH/YJM- both YPH45 and YJM789 sequences present. Graphical representation of LOH events are shown in Figure 3A and Figure S4.

### G4 insertion does not increase genome instability in WT backgrounds

**Figure 4:**
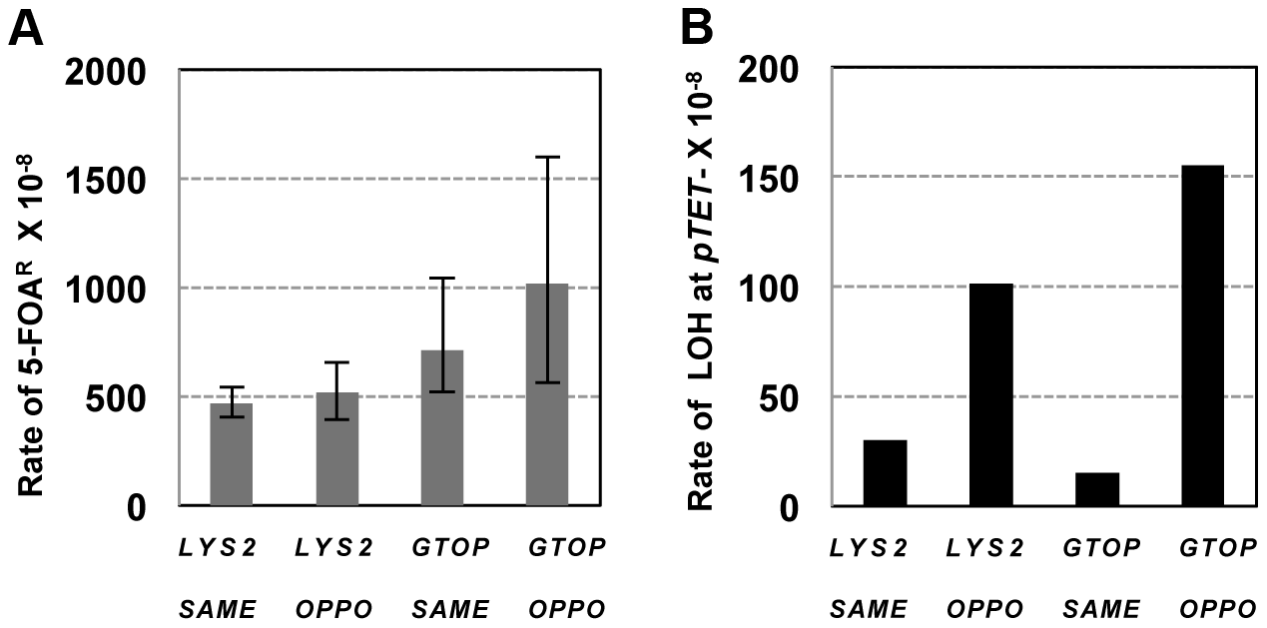
The rates of loss of heterozygosity on CHR3 in *wild-type* backgrounds. A. The total rates of LOH events (5-FOA^R^). *LYS2 or GTOP* indicates *pTET-LYS2 or pTET-lys2-GTOP* allele integrated in each of the strains analyzed. The 95% confidence intervals are indicated by the error bars. B. The rates of LOH initiating at *pTET-LYS2 or pTET-lys2-GTOP* cassette. LOH events are defined as initiating at *pTET-LYS2* or *pTET-lys2-GTOP* cassette when *URA3* and G418^R^ are lost, only YJM789 sequence is present at *Sty*I, *BsrB*I, and *Spe*I SNP sites and both YJM789 and YPH45 sequence is present at *Nar*I and *Hind*III SNP sites (See Table S2, Table 2 and Table 3). There was no statistically significant difference between all rates.

To determine whether highly transcribed G4 motifs elevate LOH on CHR3, we measured the rate of 5-FOA^R^ in WT strains containing *pTET-LYS2* (no Sμ sequence) or *pTET-lys2-GTOP* cassette. In either the SAME or OPPO orientations, the overall rate of LOH (5-FOA^R^) associated with *pTET-LYS2* or *pTET-lys2-GTOP* was not significantly different (Fig. 4A). Using the RFLP-SNP assay, we identified 3/46 or 9/46 LOH events initiated at the highly transcribed *pTET-LYS2* cassette when in the SAME or the OPPO orientation, respectively (Table 3). These proportions were not statistically different from those for LOH initiating at *pTET-lys2-GTOP* in the SAME or OPPO orientation (by Fisher's exact test; P = 0.31 and 0.39, respectively).

**Table 3:** The rates of different classes of LOH on CHR3 in *wild-type* backgrounds.

	*Strain Description* *			
LOH Class	*LYS2 SAME* Rate×10^−8^ (N)	*LYS2 OPPO* Rate×10^−8^ (N)	*GTOP SAME* Rate×10^−8^ (N)	*GTOP OPPO* Rate×10^−8^ (N)
**A**	232 (23)	112 (10)	170 (11)	333 (15)
**B**	40.4 (4)	22.4 (2)	46.3 (3)	66.5 (3)
**C**	0 (0)	0 (0)	15.4 (1)	0 (0)
**D**	20.2 (2)	33.7 (3)	61.7 (4)	111 (5)
**E** **	**30.3 (3)**	**101 (9)**	**15.4 (1)**	**155 (7)**
**F**	91.0 (9)	89.7 (8)	123 (8)	133 (6)
**G**	30.3 (3)	157 (14)	247 (16)	222 (10)
**Other**	20.2 (2)	0 (0)	30.9 (2)	0 (0)
**Total Rate×10^−8^ (95% CI)**	465 (405–546)	516 (391–653)	710 (521–1040)	1020 (563–1600)
**Total N**	46	46	46	46

LOH classes A–G are described in Table 2 and Figure S4.

*Strain Description: *pTET-LYS2 or pTET-lys2-GTOP*, SAME or OPPO replication orientation (See Fig. 3B). All data are from *TOP1 (WT)* backgrounds.

**Class E is defined as the LOH breakpoint occurring at the *pTET-LYS2 or –lys2-GTOP* cassette.

### Top1 disruption elevates mitotic recombination events initiating at a G4 motif-containing genomic locus

**Figure 5:**
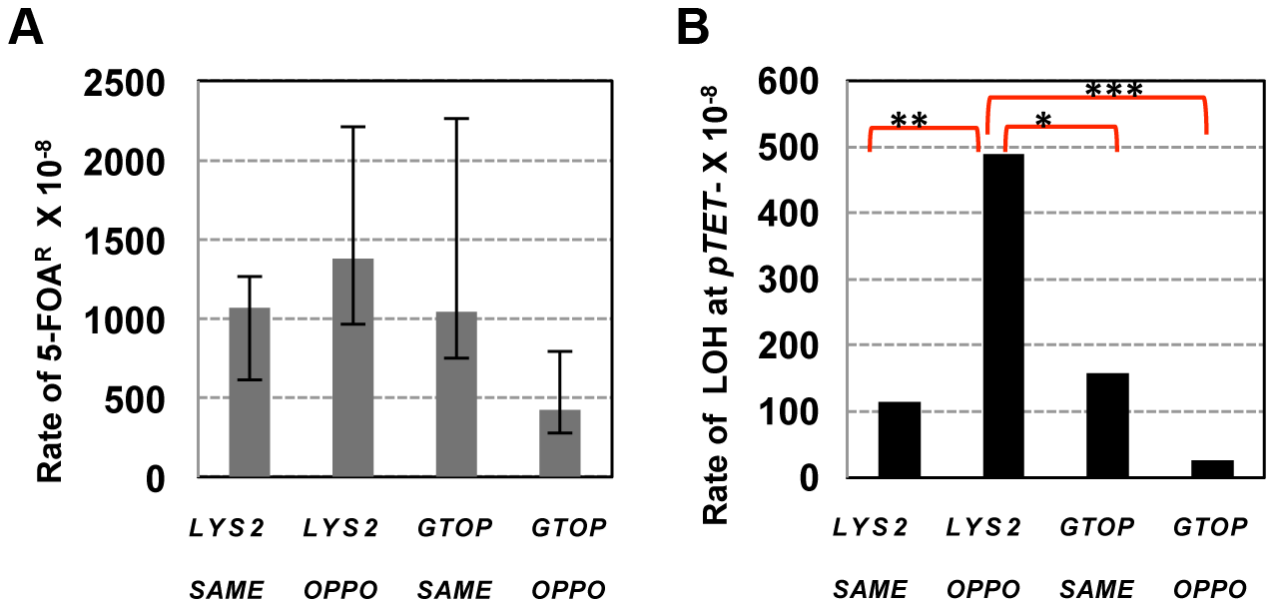
The rates of loss of heterozygosity at CHR3 in *top1*Δ backgrounds. A. The total rates of LOH events (5-FOA^R^). *GTOP, or GBTM* indicates *pTET-lys2-GTOP* or *pTET-lys2-BTM* allele integrated in each of the strains analyzed. The 95% confidence intervals are indicated by the error bars. B. The rates of LOH initiating at *pTET-lys2-GTOP* or *pTET-lys2-BTM* cassette. LOH events are defined as initiating at *pTET-lys2-GTOP* or *pTET-lys2-BTM* cassette when *URA3* and G418^R^ are lost, only YJM789 sequence is present at *Sty*I, *BsrB*I, and *Spe*I SNP sites and both YJM789 and YPH45 sequence is present at *Nar*I and *Hind*III SNP sites (See Table S2, Table 2 and Table 4). P values were determined using the chi square analysis. *P = 0.014; **P = 0.0035; ***P<0.0001.

We observed a dramatic and specific increase in the rates of gene conversion [33] and GCR (see above) associated with highly transcribed Sμ sequences when Top1 was disrupted in a haploid YPH45 background. Importantly, both occurred at significantly higher rates when the guanine-run containing strand was on the NTS where its single stranded nature fosters G4 DNA formation. In order to determine whether LOH on CHR3 is similarly affected by the location of G4-forming sequence on the TS vs. NTS, we compared LOH rates associated with *pTET-lys2-GTOP and -BTM* constructs in *top1Δ/top1Δ* backgrounds. There was no significant difference between overall rates of LOH events associated with *pTET-lys2-GTOP and -GBTM* constructs when replication was in the SAME direction (Fig. 5A). However, when replication was in the OPPO orientation, the overall rate of LOH events was ∼3 fold higher for the *pTET-lys2-GTOP* than for the *pTET-lys2-GBTM* construct. The rates of LOH initiating at the *pTET-lys2-GTOP/GBTM* cassette in the SAME or OPPO orientation in *top1Δ/top1Δ* background were determined by analyzing 47–93 5-FOA^R^ isolates by the RFLP-SNP assay (Table 4). When transcription from the *pTET* promoter was in the SAME orientation relative to replication originating at *ARS306*, the rate of LOH initiating at the G4-containing sequence was similar whether the guanine-runs were on the NTS (*pTET-lys2-GTOP*) or on the TS (*pTET-lys2-GBTM*) (Fig. 5B). However, the rate of LOH initiated near *pTET-lys2-GTOP* in the OPPO orientation was >20 fold higher than at *pTET-lys2*-GBTM in OPPO orientation and ∼4 fold higher than at *pTET-lys2-GTOP* in the SAME orientation.

**Table 4:** The rates of different classes of LOH on CHR3 in *top1Δ* backgrounds.

	*Strain Description* *			
LOH Class	*GTOP SAME* Rate×10^−8^ (N)	*GTOP OPPO* Rate×10^−8^ (N)	*GBTM SAME* Rate×10^−8^ (N)	*GBTM OPPO* Rate×10^−8^ (N)
**A**	341 (15)	134 (9)	196 (10)	194 (37)
**B**	91.1 (4)	14.8 (1)	19.6 (1)	26.2 (5)
**C**	91.1 (4)	29.7 (2)	78.5 (4)	0 (0)
**D**	22.8 (1)	44.5 (3)	19.6 (1)	36.7 (7)
**E** **	**114 (5)**	**490 (33)**	**157 (8)**	**26.2 (5)**
**F**	319 (14)	623 (42)	314 (16)	131 (25)
**G**	68.3 (3)	29.7 (2)	216 (11)	10.5 (2)
**Other**	22.8 (1)	14.8 (1)	39.2 (2)	0 (0)
**Total Rate×10^−8^ (95% CI)**	1070 (563–1600)	1380 (965–2210)	1040 (749–2260)	425 (274–796)
**Total N**	47	93	53	81

LOH classes A–G are described in Table 2 and Figure S4.

*Strain Description: *pTET-lys2-GTOP or -GBTM*, SAME or OPPO replication orientation (See Fig. 3B). All data are from *top1Δ* backgrounds.

**Class E is defined as the LOH breakpoint occurring at the *pTET –lys2-GTOP or -GBTM* cassette.

Upon Top1-disruption, when the replication and transcription is in “head-on” or OPPO orientation, the rate of loss of heterozygous SNP at *Nar*I site (at 78380 NT) is also significantly elevated (Table 4). The *Nar*I-SNP is not lost at a high rate in *pTET-lys2-GBTM*-containing strain, which suggests that recombination initiating at the *pTET-lys2-GTOP* cassette (at 68300 NT) are often associated with long conversion tracks resulting in the loss of *Nar*I-SNP. This is consistent with the average length of conversion tracks associated with reciprocal crossover events, which is reported to be about 12 kb [49].

### LOH events at G4 DNA mainly occur by crossover or break-induced replication in the absence of Top1

Using the RFLP-SNP assay described above, we characterized 93 5-FOA^R^ isolates from the *top1Δ/top1Δ* strain containing the *pTET-lys2-GTOP* cassette in the OPPO orientation and identified 33 isolates with LOH events initiating near the G4 motif-containing reporter construct (Table 4 – LOH Class E). Such LOH events can occur by (1) mitotic recombination with the other CHR3 in the heterozygous diploid cell *via* reciprocal crossover (RCO) or break-induced replication (BIR) with the homolog, (2) the partial loss of the chromosome arm, or (3) translocation to another chromosome with a short stretch of homology (Figure 3A). In order to determine the types of rearrangements occurring at this locus, separation of the CHR3 homologs by PFGE was carried out for the 33 LOH Class E isolates (Figure S7). The CHR3 homologs of YPH45 and YJM789 differ in size by about 56 kb, likely reflecting different polymorphic subtelomeric gene content and retrotransposon insertions. In 32 out of 33 5-FOA^R^ isolates analyzed, the YJM789-derived CHR3 was unchanged in size and the YPH45-derived CHR3 appeared slightly smaller than that of the parental haploid. A smaller CHR3 can be generated by RCO or BIR initiating on YPH45-derived CHR3 using YJM-789 derived CHR3 as the repair donor. The reduction in chromosome size was approximately 13 kb in 31/33 5-FOA^R^ isolates (Fig. S7). In one of 33 isolates analyzed, YPH45-CHR3 was reduced in size by ∼24 kb. An ∼8.5 kb reduction was expected from the loss of hemizygous *URA3* maker and the *pTET-lys2-GTOP* cassette with additional reduction in size resulting from the loss of other hemizygous sequences. Translocation to a heterologous chromosome was observed in 1/33 5-FOA^R^ isolates. None of the analyzed isolates contained CHR3 shortened by ∼80 kb, which is predicted in case of the loss of left arm from the *pTET-lys2-GTOP* cassette to the telomere followed by a telomere addition at the break site (Figure 3A, bottom panel).

### Replication-orientation significantly affects gene conversion rates associated with G4-forming sequence

**Figure 6:**
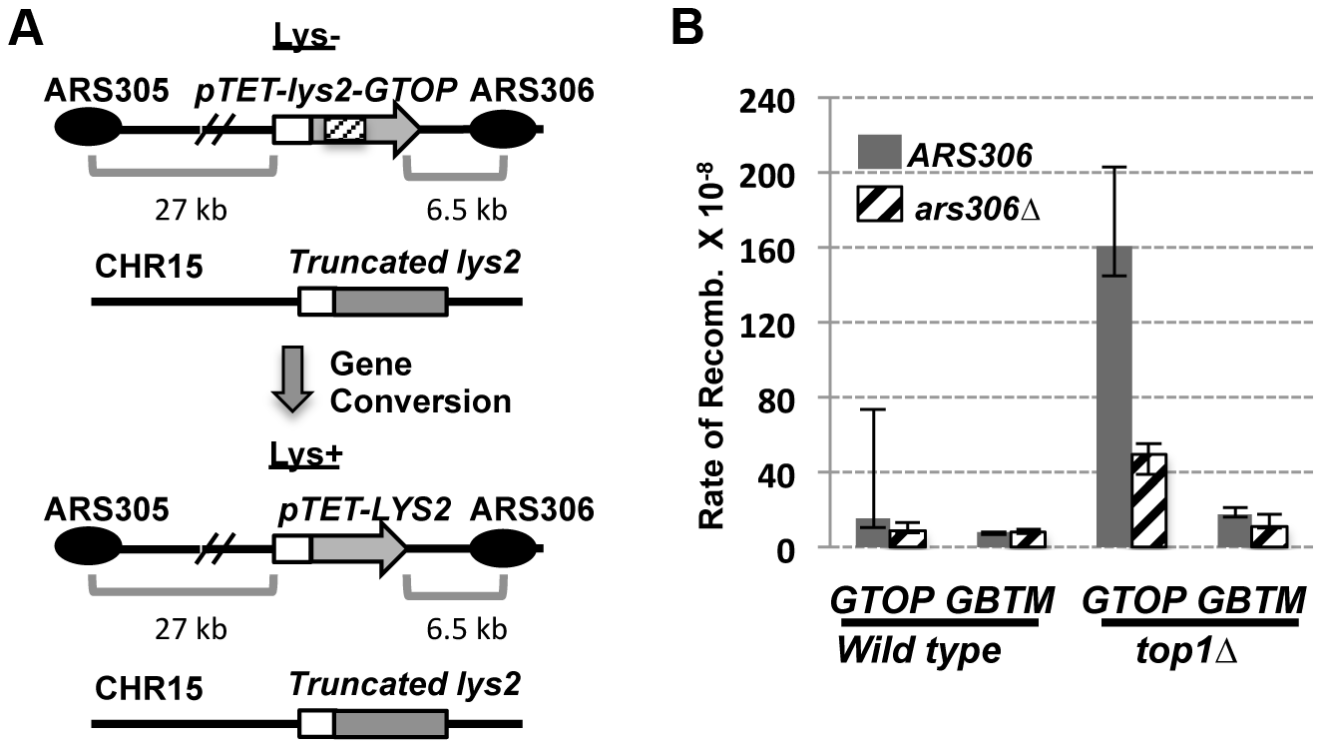
The effect of replication direction on the gene conversion rate. A. A schematic representation of the gene conversion assay. The *pTET-lys2-GTOP* cassette (indicated by the hashed box) is located in “Head-on” orientation relative to *ARS306*. In the *ars306Δ* strain, the closest origin of replication is *ARS305* from which replication proceeds in a co-directional orientation with the transcription from *pTET*. The distances (in kb) are approximate and not to scale. B. The rates of gene conversion at *pTET-lys2-GTOP* or –*GBTM* cassette. Transcription and replication are in the “Head-on” orientation when *ARS306* (gray bar) is present and in the “Co-directional” orientation in *ars306Δ* strains (hashed bar). The 95% confidence intervals are indicated by the error bars.

In our previous report regarding the rate of gene conversion events induced by the highly transcribed Sμ sequence, the *pTET-lys2-GTOP* cassette was integrated in the orientation and location identical to the “OPPO” construct described above for the LOH assay (Fig. 6A). In this orientation, transcription from *pTET* promoter and replication originating at *ARS306* are in the convergent or “head-on” orientation. In order to determine whether the rate of gene conversion is dependent on the relative orientation of transcription and replication, we deleted *ARS306* by replacing the ARS consensus sequence (5′-WTTTAYRTTTW-3′) [50] with the gene encoding hygromycin B phosphotransferase (Hph). In the resulting *ars306Δ* strain, replication through the *pTET-lys2-GTOP* cassette originates from the *ARS305* located about 27 kb away and is in co-directional orientation relative to transcription [51]. As previously reported, in the gene conversion assay, recombination initiating at the *pTET-lys2* cassette can be completed using a truncated *lys2* gene fragment integrated on CHR15 resulting in lysine prototrophy (Lys^+^) (Fig. 6A) [33]. In a *top1Δ* strain containing the *pTET-lys2-GTOP* cassette, reversing the replication orientation by deletion of *ARS306* resulted in a three-fold decrease in the Lys^+^ rate indicating that replication-transcription conflict is a factor in elevated gene conversion initiating at co-transcriptionally formed G4 DNA (Fig. 6B). The deletion of *ARS306* did not significantly affect the gene conversion rates at *pTET-lys2-GBTM* in *top1Δ* background or at *pTET-lys2-GTOP* or –*GBTM* cassette in *WT* backgrounds.

## Discussion

G4 motifs have been implicated in various types of genome instability events. However, the large number of sequences predicted form G4 DNA structures have not all been validated as potential hotspots of genome rearrangements. We here focused on the level of transcription as a singularly important genomic context that distinguishes genetically unstable G4-forming sequence. At the Ig heavy chain locus, class-switch recombination (CSR) in activated B cells requires switch regions consisting of long repetitive sequences dense with guanine-runs [52]. In the pathogenic bacteria *Neisseria gonorrhoeae*, a G4 DNA-forming sequence was identified to be essential for the gene conversion occurring at the *PilE* locus, which facilitates evasion of host adaptive immune system by the production of variant pilin subunits [53]. Transcription of the guanine-run containing sequences is required to initiate recombination in both of these processes [54], [55]. One possible role played by transcription is to provide the strand separation necessary for guanine-runs to fold into G4 structures. In order to determine the effect of G4 DNA on yeast genome stability, we designed our genetic assays to assess the role of transcription in biological processes. For effective formation of G4 DNA during transcription, we placed a model G4 motif from the mouse Ig switch Mu region into the highly transcribed *pTET-LYS2* cassette. The guanine-runs were placed on non-transcribed strand (*-GTOP*) to promote co-interaction in single-strand context; as a negative control, genomic instability associated with the same G4 sequence was measured when transcribed in inverse orientation (-*GBTM*) where guanines on the TS interact with the nascent RNA and are not available for G4 formation (Fig. 1B).

In the gene conversion assay we used previously, only those recombination events initiating specifically at the reporter construct could be phenotypically selected [33]. In the current study, we designed two other genomic assays that allowed us to survey instability initiated over large genomic areas that includes our G4 motif-containing reporter construct. Loss of part of a chromosome arm initiating over a ∼20 kb region of CHR5 in a haploid background (GCR assay) or recombination initiating over a ∼100 kb region of CHR3 in a diploid background (LOH assay) yielded selectable, Can^R^ 5-FOA^R^ or 5-FOA^R^ colonies, respectively (Fig. 1A and 3A). By further analysis of the individual isolates, we were able to estimate the locations of GCR or mitotic recombination initiation. Importantly, this unbiased measurement of spontaneous genome rearrangements enabled us to define the conditions under which GCR and mitotic recombination resulting in LOH are specifically elevated at the highly transcribed G4 motif.

Eukaryotic Top1 has multiple functions during DNA transactions [56]. Both negative and positive supercoils accumulated during transcription are removed by Top1 activity. Together with the type II topoisomerase Top2, Top1 is recruited to the genomic regions undergoing replication [57] and has a role in relieving transcription-replication conflicts [40]. Top1 function can also adversely affect genome stability; its endo-ribonuclease activity generates unligatable single-strand breaks at ribonucleotides embedded in DNA, which leads to replication stress and accumulation of deletion mutations [58], [59]. We have shown here that, at the co-transcriptionally generated G4 DNA, Top1 activity is required to suppress various types of genome instability. Top1 disruption greatly elevated overall GCR rates when the *pTET-lys2-GTOP* was present on CHR5, with 90% of GCR breakpoints mapping to the G4 DNA-forming sequence (Fig. 1C and Table 1). This elevation was completely dependent on the level of transcription and the location of the guanine-runs on the NTS, reinforcing the conclusion that a potential G4 sequence motif is transformed by transcription into a genome instability hotspot. In the LOH assay, even though the overall mitotic recombination rate was not considerably elevated in the *top1Δ/top1Δ* background, a significantly higher proportion of the LOH tract breakpoints mapped to the *pTET-lys2-GTOP* but not to *the pTET-lys2-GBTM* cassette within the left arm of CHR3 (Fig. 5 and Table 4).

In absence of Top1, the rates of both gene conversion and LOH occurring at the G4-forming sequence were significantly higher when the transcription was in “head-on” or collisional orientation with replication fork movement than when it was in co-directional orientation (Figure 5B and 6B). This suggests that Top1-dependent suppression of G4-associated genome instability involves its activity of resolving transcription-replication conflict in addition to its activity of resolving transcription-associated torsional stress. This also suggests that, in addition to the level of transcription, the relative orientation of replication is an important genomic context that can render certain G4 motifs genetically unstable. Accumulation of stalled replication forks at gene-rich regions have been observed in Top1-deficient cells indicating that one of the ways Top1 prevents genome instability is to prevent replication fork collapse due to collision with transcription [40]. Alternatively, the significantly higher LOH and gene conversion rates observed when the transcription and replication are in “head-on” orientation can be due to the intrinsic asymmetry in the replication process. In “head-on” or co-directional orientation, G-runs are present in the leading strand or lagging strand, respectively. It is possible that, upon encountering G4 DNA, the lagging strand synthesis is less prone to replication arrest since re-priming downstream will allow continued replication fork movement. It was previously reported that replication orientation did not have an effect when the G4-forming human subtelomeric minisatelite CEB1 was placed into the yeast genome [30]. The rates of GCR at this G4 forming sequence were elevated to similar degrees whether the G-runs were on the leading strand or lagging strand. In this experiment, CEB1 was not transcribed, supporting the argument that the orientation bias we observed with the highly transcribed Sμ is due to conflict between replication and transcription.

In cultured mouse B cells, it was reported that class switch recombination (CSR) at Ig heavy chain locus was inhibited by camptothecin (CPT) treatment and significantly elevated by siRNA-mediated knock-down of Top1 [60]. It was suggested that G4 DNA formation is facilitated by reduced Top1 activity and that DNA ligation by Top1, which requires the proper alignment of 3′ and 5′ ends of the breaks, is inhibited by interaction with DNA secondary structures resulting in Top1 cleavage complex and unresolved DNA breaks that initiate CSR. However, we demonstrated here that genome instability associated a G4 motif is stimulated by the complete absence of the Top1 protein (*top1Δ*) (Fig. 1C, 5B and ref. [33]). DNA breaks initiating gene conversion, LOH or gross chromosomal rearrangements may originate from other sources such as G4-specific nucleases or from collapsed DNA replication forks.

Multiple helicases including human FANCJ, PIF1, BLM and yeast Sgs1 can unwind G4 structures *in vitro* [61], [62]. In BLM-deficient cells, G4 motifs are frequently found near the transcription start sites of those genes with perturbed expression profile suggesting a role of BLM helicase in G4-mediated gene regulation [63]. Using GCR assays similar to that described above, two independent investigations into the effect of mutation of the 5′-to-3′ DNA helicase Pif1 on the rate of GCR initiated by G4-forming sequences reported dramatically disparate results [30], [61]. Although greatly increased rates of FOA^R^/Canavanine^R^ colonies were observed in both investigations, further analyses revealed that the 5-FOA^R^/Canavanine^R^ colonies in yeast cells expressing mutant Pif1 arose mostly *via* rearrangements or partial loss of the chromosome in one case [30] but mainly *via* epigenetic silencing of the *CAN1* and *URA3* genes in another [61]. In case of the G4-associated GCR events occurring due to Top1-deficiency reported here, we demonstrated that the simultaneous resistance to 5-FOA and canavanine resulted from the loss of the region of the CHR5 containing *CAN1* and *URA3* genes (Table 1). In 60% of the GCR events involving the highly transcribed G4 motif, *de novo* telomere addition occurred within the guanine-run containing Sμ region (Fig. 2 and Fig. S1). Other types of events included 15 kb segmental deletions and complex genome rearrangements involving terminal deletions and segmental duplications (Fig. 2B. Fig. S2 and Fig. S3). When combined with co-transcriptionally formed G4 DNA, Top1 disruption significantly reshapes the genome not just through elevated non-crossover and allelic interhomolog recombination but also through gross deletions and duplications resulting in copy number variations. This result suggests that, whereas the function of Pif1 and BLM is possibly linked to the role of G4 DNA as an epigenetic and transcriptional regulator [17], [61], [63], Top1 functions directly to prevent chromosomal rearrangements and gross loss of genetic information associated with the G4 DNA, particularly at highly transcribed areas.

**Figure 7:**
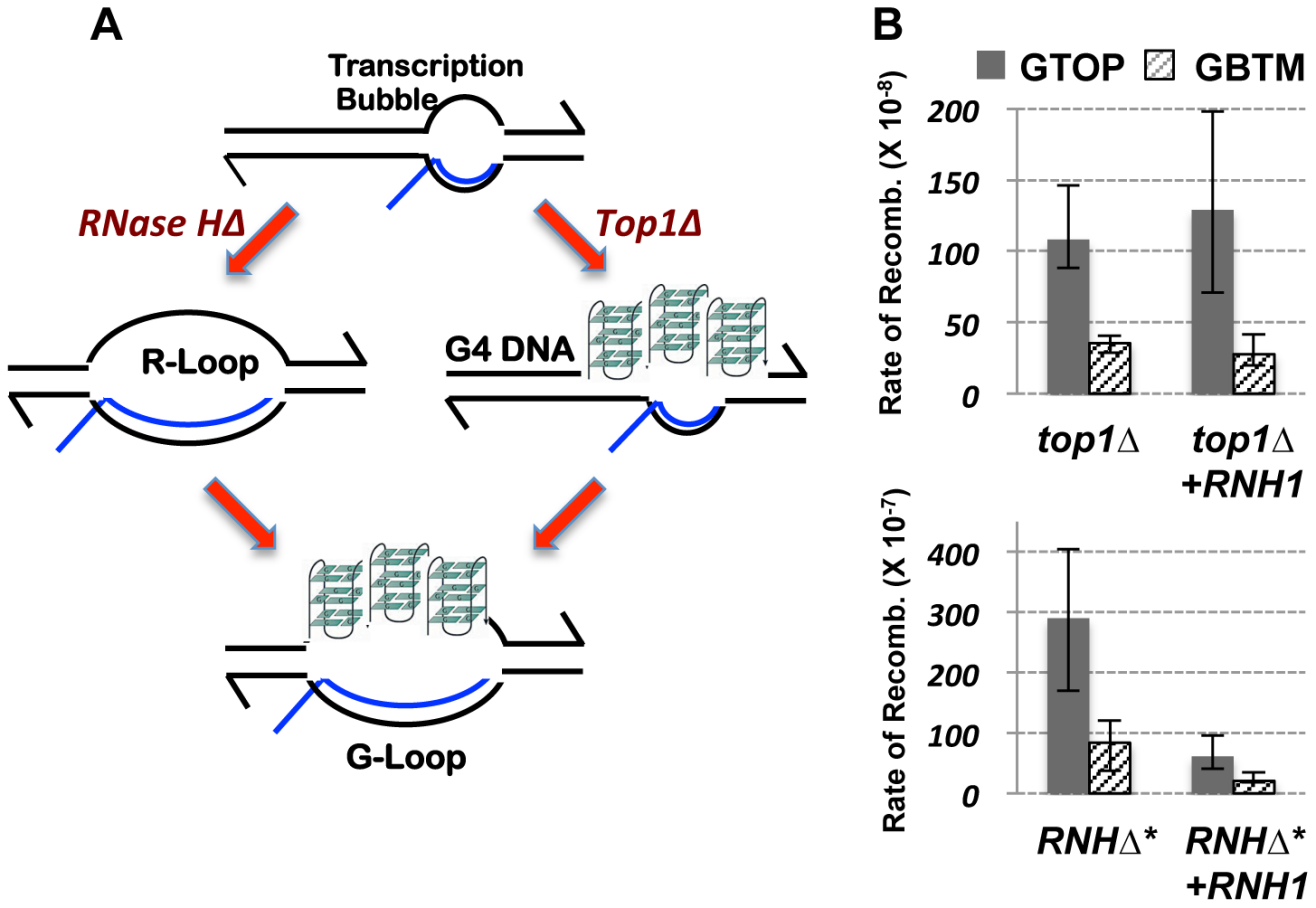
The RNA∶DNA hybrid and gene conversion at the highly transcribed Sμ. A. A model depicting two alternate pathways of G-loop formation. In absence of RNase H activity, RNA∶DNA hybrid accumulation fosters folding of G-runs into G4 DNA. In absence of Top1 activity, G4 DNA, which function as a sink for negative torsional stress, can promote RNA∶DNA hybrid formation. B. The rates of gene conversion at *pTET-lys2-GTOP* (gray bar) or –*GBTM* (hashed bar) cassette. Indicated strains were transformed with either pGAL1-RNH1 (+RNH1) or pRS416 as vector control. RNHΔ* indicates *rnh1Δ rnh201Δ* double mutant strains. The 95% confidence intervals are indicated by the error bars.

Activated transcription through G/C rich sequence can lead to formation of R-loops, which comprise of a long and stable hybrid between nascent RNA and template DNA strand [64]. R-loop accumulation and associated hyper-recombination can ensue when the mRNA packaging and export is disturbed in THO-TREX defective strains or when the degradation of RNA in RNA∶DNA hybrid is deficient due to absence of RNase H activity [65]. Accumulation of negative supercoils in Topoisomerase-deficient cells can also lead to R-loop accumulation [66]. Duquette et al reported that the combination of R-loop and G4 DNA, referred to as G-loop, is identifiable by electron microscopy when the Ig switch sequence is highly transcribed either *in vitro* or in bacteria [13]. At the *pTET-lys2-GTOP* cassette containing the Sμ sequence, therefore, the elevated genome instability could be the result of RNA∶DNA hybrid and/or G4 DNA. G-loop formation can be instigated by G4 DNA nucleation in the NTS, which lead to the stable annealing of the nascent RNA with the unpaired TS of DNA (Fig. 7). Alternatively, G-loop formation can initiate *via* the formation of RNA∶DNA hybrid, which leaves the NTS unpaired and free to fold into G4 structure. In this case, the higher stability of rG∶dC base pairing compared to rC∶dG could account for the greater instability we observed when the G-runs are on the NTS (*-GTOP*) [67]. During *in vitro* transcription, rG∶dC containing RNA∶DNA hybrid is critical for the formation of G-loop structure by Ig switch sequence [13], and required for the transcription blockage by a guanine-run [19]. We tested whether G4-induced hyper-recombination is dependent on RNA∶DNA hybrid formation by overexpressing RNase H1 in *top1Δ* background. RNase H1 is an enzyme that degrades RNA hybridized to DNA and was shown to counteract the hyper-recombination phenotype associated with R-loops in THO/TREX mutant background [65]. As shown in Fig. 7B, RNase H1 overexpression did not reduce the elevated recombination at the highly expressed *pTET-lys2-GTOP* cassette, which suggests that RNA∶DNA hybrid is not required for the elevated recombination occurring at the G4 motif in absence of Top1. We previously reported that, upon disruption of both RNase H1 and RNase H2 (*rnh1Δ rnh2Δ*), the rates of gene conversion for the *pTET-lys2-GTOP* and *–GBTM* constructs were elevated by 28- and 8- fold, respectively [33]. RNase H1 overexpression led to significant decreases in the rates of gene conversion in *rnh1Δ rnh2Δ* backgrounds indicating that RNA∶DNA hybrid is responsible for the elevated recombination at both the *pTET-lys2-GTOP* and *–GBTM* constructs in *rnh1Δ rnh2Δ* mutant strains (Fig. 7B). We postulate that RNA∶DNA hybrid and G4 DNA can each result in genome instability but that R-loop is not the primary cause of elevated recombination we observed for the *pTET-lys2-GTOP* upon disruption of Top1. A function of Top1 other than the prevention of R-loop formation is relevant in suppressing genome instability at G4 DNA, which will require further investigation to identify.

In summary, we report here the identification of Top1 as an important factor in suppressing genome rearrangements instigated by co-transcriptionally formed G4 DNA. In the absence of Top1, LOH-inducing mitotic recombination as well as GCR is highly elevated but only when the guanine-run containing sequence is located on the NTS and is highly transcribed. The exquisitely specific effect of Top1 at G4 DNA is underscored by recent reports that, without co-transcriptionally formed G4 DNA, Top1-disruption had no significant effect on GCR or gene conversion rate and even suppressed the GCR or gene conversion occurring in cells with defects in the ribonucleotide excision repair (RER) pathway [68], [69]. One possible explanation for its functional specificity is the high affinity binding of G4 DNA by Top1 demonstrated in *in vitro* experiments [70]–[72]. Top1 activity in suppressing G4-assoicated genome instability becomes even more important when transcription is in the collisional orientation with replication. This result suggests that, besides the transcription-conferred strand bias, the genomic location relative to a replication origin might determine which of the numerous G4 motifs so far identified in the eukaryotic genomes might be a hotspot of genome instability. The data presented in this report opens up the possibility that other factors suppressing G4-associated genome instability will be found among proteins with known physical and/or genetic interactions with Top1.

## Materials and Methods

### Yeast strains

Yeast strains used for the GCR assay and the gene conversion assay were derived from YPH45 (*MATa, ura3-52 ade2-101 trp101*; [73]). For construction of the GCR assay, procedures for deletion of endogenous *LYS2* on chromosome II and insertion of tetR′-SSN6 repressor-expressing cassette at *LEU2* locus (pCM244 from Euroscarf [74]) were as described previously for the gene conversion assay [33]. A PCR-amplified *LYS2* gene fragment was then integrated upstream (centromere proximal) of the *CAN1* ORF on the left arm of chromosome V. The *LYS2* promoter was replaced with a PCR-generated cassette from pCM225 (Euroscarf) containing the *pTET* promoter with 7 repeats of tetO and the tetR-VP16 activator coding sequence. The replacement of *LYS2* with either the *lys2-GTOP* or -*GBTM* allele was carried out using the two-step allele replacement method. Finally, the loxP-flanked *URA3Kl* cassette was amplified from pUG72 (Euroscarf; [75]) to replace the *HXT13* gene through one-step allele replacement. The construction of *pTET-LYS2* and *pTET-lys2-GTOP* or -*GBTM* (previously referred to as *pTET-lys2-SμF* and *–SμR*) cassettes and the genomic integration on chromosome III in the YPH45 strain background were previously described [33], [36]. For the loss of heterozygosity (LOH) assay, the YPH45-derived haploids were mated to an YJM789-derived haploid (*MATα, ura3 lys2*; [48]).

The plasmid pGAL1-RNH1 with the yeast *RNH1* gene under the galactose-inducible *GAL1* promoter was a gift from R. Crouch (NCI; Bethesda, MD).

### Determination of Rates

For the GCR assay, 5 ml cultures in YEPD medium (1% yeast extract, 2% Bacto-peptone, 2% dextrose, and 250 µg/mL adenine hemisulfate, 2% agar for plates) were inoculated with single colonies and grown for 3 days at 30°C. Cells were then plated either on YEPD or synthetic complete dextrose medium lacking arginine (SCD-arg) and containing canavanine (60 mg/L) and 5-Fluoroorotic acid (5-FOA; 1 g/L). For the LOH assay, 1 ml YEPD cultures inoculated with single colonies were grown for 3 days at 30°C and plated on YEPD or SCD containing 5-FOA (1 g/L). For determination of gene conversion rates, growth and plating conditions were the same as previously described [33]. For *RNH1* overexpression experiment, indicated yeast strains were transformed with pGAL1-RNH1 plasmid or pRS416. Individual Ura^+^ transformants were used to inoculate 1 ml cultures in SCD-Ura media supplemented with 1% raffinose and 2% galacotse. After 4 days growth at 30°C, appropriate dilutions of the cultures were plated on YEPD or SCD-Ura-Lys. For each strain, 12 to 36 cultures were used to determine rates and 95% confidence intervals using the Lea-Coulson method of median [76], [77]. Where indicated, rates were determined using the *p*_0_ method [78].

### Molecular karyotype analysis of gross chromosomal rearrangements (GCRs)

Characterization of GCR events using pulse field gel electrophoresis (PFGE) and microarray-based comparative genome hybridization (array-CGH) were carried out as previously described [79]. The microarrays used were Agilent custom 8x15k design (AMID 028943), with 14,965 unique 60 nt oligonucleotide probes, and a median genomic spacing of 774 bp. Detailed microarray probe composition and hybridization data are available upon request.

### Sequencing the inverted duplication breakpoints of Class III GCRs

The *Bgl*II-digested pAG25 plasmid [80] was integrated at a site proximal to the KanMX4 marker present in the class III clones. Genomic DNA from the clones containing the integrated plasmid was extracted and digested with *Eco*RV followed by re-ligation and rescue of re-circularized plasmids in *E. coli*. Restriction analyses of the rescued plasmids were consistent with inverted duplication structures. *Sac*I restriction fragments containing the center of symmetry of the inverted duplicated regions were sub-cloned into a pUC18 plasmid vector and sequenced using primers positioned just outside the vector's multicloning site. The sequences of the inverted duplication breakpoints from clones A7 and A8 are shown in Fig. S4A and S4B. The secondary chromosomal rearrangements in class III had breakpoints at Ty/LTR sequences, and were consistent with the ectopic homologous recombination mechanism most often observed in yeast GCRs [81].

## Supporting Information

Figure S1 Sequence analysis of 5-FOA^R^ Can^R^ isolates with *de novo telomere addition at Sμ.* The Sμ sequence in unperturbed *pTET-lys2-GTOP* cassette is in bold/capital letters at the top (Sμ). Base changes are indicated by the lower case letters. ****; the start of telomere sequence. Δ; deletions, +; insertions, ///; segmental duplications.
(PDF)

Figure S2 Molecular karyotype analysis of CHR5 GCRs. A. GCR class I, clone A13; B. GCR class II, clone A6; C. GCR class IIIa, clone A16; and D. GCR class IIIb, clone A7. In all panels, the top part corresponds to sections of the same PFGE gel shown in Fig. 2A, cropped between the CHR13/6 and CHR9 regions. Directly above the PFGE images are the superimposed plots of the quantitative trace analysis of the PFGE lanes for the parental strain (dark blue trace) and for the respective GCR clone (red trace). The image pixel intensity traces (vertical axis) for the GCR PFGEs were normalized relative to the trace of the parental strain. The parental and GCR traces closely overlapped for all chromosomes other than CHR5 and the rearranged CHR5 in the various GCR classes. The approximate size of CHR5 and the GCRs are indicated. The lower part of each panel shows the array-CGH copy number plots for the probes (blue dots) from CHR5 in the respective GCR clones. The vertical axis corresponds to the Log2(GCR[Cy5]/Parent[Cy3]) signal and the corresponding positions for 0, 1, and 2 copies of genomic material in the respective GCR clones. The horizontal axis corresponds to the physical position of each probe on CHR5. Regions where full deletions where detected are shaded in red, and regions where duplications were detected are shaded in light blue. The specific breakpoints in each GCR class are summarized in main text and in Fig. 2B.
(PDF)

Figure S3 The direct repeat-mediated deletions in Class II GCR events. A. The locations of the 21 nt direct repeats (underlined) before the deletion are shown relative to the relevant genetic features. Directions of transcription for genes listed are shown with red arrows. DR1 originated from pUG72 plasmid used to generate the *loxP-URA3Kl-loxP* cassette, which replaced the *HXT13* ORF. DR2 originated from pCM225 plasmid used to replace *pLYS2* with *pTET*. B. The genomic configuration after the 15 kb deletion between the 21 nt direct repeats. C. The sequences flanking the 21 nt sequence after the 15 kb deletion (in lower case letters) were confirmed by PCR-sequencing to be from *HXT13*-proximal (in red) and *pTET*-proximal (in blue).
(PDF)

Figure S4 Fold-back inverted duplications originating at the G-quadruplex sequence. A and B. The DNA sequence determined from the inverted duplication structures in clone A7 (A) and A8 (B) are shown at the bottom of each figure (Step 5), and the proposed mechanism of formation is shown above them. Step 1. A DNA double strand break forms within or proximal to the Sμ sequence. Step 2. 5′ to 3′ resection creates a ssDNA region. Step 3. The 3′ end of the ssDNA folds back on itself forming a loop of 59 nt (A – clone A7) or 441 nt (B – clone A8) (nucleotides in green) and anneals to a 4 nt microhomology (underlined). Step 4. The annealed 3′ end primes break-induced replication (nucleotides in blue) going toward the right arm. Step 5. After BIR, a complementary DNA strand is synthesized (nucleotides in red) to complete the inverted chromosomal rearrangement event. Regions deleted and duplicated in the resulting clone are shaded in red and blue, respectively. The arrows above and below the DNA sequence show the two sides of the inverted duplication, which flank the microhomologies and the single copy region corresponding to the original ssDNA loop. C. Models for the generation of the recovered Class III GCRs. The inverted duplication rearrangements initiated by a double strand break lesion at the Sμ sequence. Unique regions in CHR5 are labeled with letters A through F. The CHR5 fragment to the left of Sμ containing *TEL05L*, *URA3*, and *CAN1* is lost (segment A), while the resected 3′ end of the CHR5 fragment to the right of Sμ folds back on itself and initiates BIR at the B segment. At this point, two possible models are shown to explain the structure of the recovered clones. Model 1: Template switching. 1A. BIR proceeds from left to right through the B and C segments (green line) until reaching a dispersed Ty or LTR repeat (open white arrow). 1B. At this point the BIR fork collapses, and re-anneals at a homologous Ty or LTR on the right arm, then resumes extension copying the F segment (orange line) until reaching the right telomere. The resulting GCR product has a deletion of the A segment, an inverted duplication of B and C, single copy of D and E (including the centromere), and a duplication of F. Model 2: Dicentric formation and secondary rearrangement. 2A. BIR copies an uninterrupted template through segments B, C, and D, then across the centromere, then through segments E and F reaching the right telomere (pink line). 2B. Since the resulting GCR is dicentric, it inevitably accumulates a new DNA break that leads to a secondary rearrangement between directly oriented Ty or LTR repeats flanking one of the two centromeres (dashed double-ended arrow). The resulting secondary GCR becomes monocentric and is recovered as a viable Class III clone.
(PDF)

Figure S5 Reciprocal crossover (RCO) and break induced replication (BIR) producing 5-FOA^R^ recombinants. A simplified diagram depicting RCO or BIR between the heterozygous CHR3s in LOH assay (see Fig. 3). YPH45-derived or YJM789-derived CHR3s are depicted in black or red lines, respectively. Centromeres are represented with gray circle in each diagram. The diploid cell in S-phase with duplicated CHR3s is shown at the top. After repairing DNA breaks by RCO or BIR, two progenies (one 5-FOA^R^ and one 5-FOA^S^) produced after chromosome segregation and cell division are depicted at the bottom. BIR and RCO will produce one progeny containing one copy or two copies of *URA3* (shaded in gray), respectively, which will not grow on the 5-FOA-containing selection media. The 5-FOA^R^ progenies from RCO and BIR (not shaded) are identical. Because we are unable to analyze the 5-FOA^S^ product of the mitosis, we cannot distinguish whether the recombinant CHR3s observed are products of RCO or BIR, which produce identical 5-FOA^R^ daughters.
(PDF)

Figure S6 **LOH classes not initiated at the** *pTET-lys2-GTOP* or *-GBTM* cassette. Schematic representations of mitotic recombination products resulting in the selected the 5-FOA^R^ LOH events are shown. LOH classe E initiated at the *pTET-lys2-GTOP* or *-GBTM* cassette are shown in Figure 3A. YPH45-CHR3 and YJM789-CHR3 are represented by black and red lines, respectively. Location of the *pTET-lys2-GTOP* or *–GBTM* cassette on the left arm of CHR3 is indicated by the hashed box. Green boxes indicate the approximate locations of the heterozygous SNP markers listed in Table S2. Telomeres are represented as “tgtgtg”. Hemizygous *URA3* and *G418^R^* markers present only on the YPH45-CHR3 homolog are indicated by blue boxes. LOH classes A–G are defined by RFLP-SNP assay. Briefly, SNPs between the YJM789 and YPH45 CHR3 sequences were identified by comparing the genome sequences (Saccharomyces Genome Database (SGD). Then, five sites were chosen where the SNP either generates or abolishes a restriction enzyme recognition site. At a location equivalent to the SGD coordinate 54440 in S288c, for example, a *Sty*I cut site (CCAAGG) is present on YPH45-derived CHR3. On YJM789 CHR3, this sequence differs by one nucleotide (CCAAGA), and cannot be cut by *Sty*I. In a diploid carrying both copies of CHR3 with this region unchanged (heterozygous), PCR amplification followed by *Sty*I digestion will detect both cut (YPH45) and uncut (YJM789) alleles. If a recombination event in the heterozygous diploid results in the loss of this region of YPH45-CHR3, only the uncut YJM789 fragment will be present. Additionally, we tested by PCR whether the hemizygous *URA3* and G418^R^ markers, were retained in the 5-FOA^R^ isolates. All sites surveyed with the RFLP-SNP assay are listed in Table S2.
(PDF)

Figure S7 PFGE analysis of LOH (5-FOA^R^) isolates. YPH45/YJM789 hybrid CHR3 with size estimated at 362 kb is indicated with the red asterisk. Parental haploids YPH45 and YJM789 are each indicated. Lanes F#2–F#19 are 5-FOA^R^ diploid isolates derived from the strain containing *pTET-lys2-GTOP (OPPO)* cassette in *top1Δ* background.
(PDF)

Table S1 The relative RNA levels of *lys2-GTOP* or -*GBTM* allele in *top1Δ* backgrounds.
(PDF)

Table S2 SNPs or hemizygous markers used for RFLP-SNP assay.
(PDF)
