# Supplementary material for: Topoisomerase I Plays a Critical Role in Suppressing Genome Instability at a Highly Transcribed G-Quadruplex-Forming Sequence
Source: PLoS Genet. 2014 Dec 4;10(12):e1004839. doi: 10.1371/journal.pgen.1004839 (PMC4256205; doi:10.1371/journal.pgen.1004839)
Supplement: Figure S1 — Sequence analysis of 5-FOAR CanR isolates with de novo telomere addition at Sμ. The Sμ sequence in unperturbed pTET-lys2-GTOP cassette is in bold/capital letters at the top (Sμ). Base changes are indicated by the lower case letters. ****; the start of telomere sequence. Δ; deletions, +; insertions, ///; segmental duplications. (PDF) [file pgen.1004839.s001.pdf]

```
              10         20         30         40         50         60         70         80         90         100
Sμ    GATCT GAGCTGAGCT GAGCTGGGTG AGCTGAGCTG AGCTGAGCTG GGTGAGCTGA GCTGAGCTGA GCTGAGCTGG GTGAGCTGAG CTGAGCTGAG CTGAGCTGAG
A1    ----- ---------- ---------- ---------- ---------- ---------- ---------- ---------- ---------- ---------- ----------
A30   ----- ---------- ---------- ---------- ---------- ---------- ---------- ---------- ---------- ---------- ----------
A10   ----- ---------- ---------- ---------- ---------- ---------- ---------- ---------- ---------- ---------- ----------
A2    ----- ---------- ---------- ---------- ---------- ---------- ---------- ---------- ---------- ---------- ----------
A25   ----- ---------- ---------- ---------- ---------- ---------- ---------- ---------- ---------- ---------- ----------
A13   ----- ---------- ---------- ---------- ---------- ---------- ---------- ---------- ---------- ---------- ----------
A18   ----- ---------- ---------- ---------- ---------- ---------- ---------- ---------- ---------- ---------- ----------
A27   ----- ---------- ---------- ---------- ---------- ---------- ---------- ---------- ---------- ---------- ----------
A3    ----- ---------- ---------- ---------- ---------- ---------- ---------- ---------- ---------- ---------- ----------
A21   ----- ---------- ---------- ---------- ---------- ---------- ---------- ---------- ---------- ---------- ----------
A12   ----- ---------- ---------- ---------- ---------- ---------- ---------- ---------- ---------- ---------- ----------
A20   ----- ---------- ---------- ---------- ---------- ---------- ---------- ---------- ---------- ---------- ----------
A26   ----- ---------- ---------- ---------- ---------- ---------- ---------- ---------- ---------- ---------- ----------
A4    ----- ---------- ---------- ---------- ---------- ---------- ---------- ---------- ---------- ---------- ----------
A23   ----- ---------- ---------- ---------- ---------- ---------- ---------- ---------- ---------- ---------- ----------

         110        120        130        140        150        160        170        180        190        200        210
Sμ    TGAGCTGGGGTGAGCTGGG CTGAGCTGAG CTGAGCTGGG GTGAGCTGAG CTGAGCTGAG CTGAGCTGGG GTGAGCTGGG CTGAGCTGGG GTGAGCTGGG CTGAGCTGAG
A1    -------------------- ---------- ---------- ---------- ---------- ---------- ---------- ---------- ---------- ----------
A30   -------------------- ---------- ---------- ---------- ---------- ---------- ---------- ---------- ---------- ----------
A10   -------------------- ---------- ---------- ---------- ---------- ---------- ---------- ---------- ---------- ----------
A2    -------------------- ---------- ---------- ---------- ---------- ---------- ---------- ---------- ---------- ----------
A25   -------------------- ---------- ---------- ---------- ---------- ---------- ---------- ---------- ---------- ----------
A13   -------------------- ---------- ---------- ---------- ---------- ---------- ---------- ---------- ---------- ----------
A18   -------------------- ---------- ---------- ---------- ---------- ---------- ---------- ---------- ---------- ----------
A27   -------------------- ****
A3    -------------------- ---------- ---------- ---------- ---------- ---------- ---------- ---------- ---------- ----------
A21   -------------------- ---------- ---------- ---------- ---------- ---------- ---------- ---------- ---------- ----------
A12   -------------------- ---------- ---------- ---------- ---------- ---------- ---------- ---------- ---------- ----------
A20   -------------------- ---------- ---------- ---------- ---------- ---------- ---------- ---------- ---------- ----------
A26   -------------------- ---------- ---------- ---------- ---------- ---------- ---------- ---------- ---------- ----------
A4    -------------------- ---------- ---------- ---------- ---------- ---------- ---------- ----a---a- c--------- Δ---------
A23   -------------------- ---------- ---------- --------g- ---------- ---------- --------a- ---------Δ --------a- ----------

         220        230        240        250        260        270        280        290        300        310        320
Sμ    TGGGTGAGC TGAGCTGAAC TGAGCTGAGC TGGGTGAGCT GAGCTGAGCT GAGCTGGGTG AGCTGAGCTG GGCTGAGCTG AGCTGGGTGA GCTGAGCTGA ACTGAGCTGA
A1    -------------------- ---------- ---------- ---------- ---------- ---------- ---------- ---------- ---------- ----------
A30   -------------------- ---------- ---------- ---------- ---------- ---------- ---------- ---------- ---------- ----------
A10   -------------------- ---------- ---------- ---------- ---------- ---------- ---------- ---------- ---------- ----------
A2    -------------------- ---------- ---------- ---------- ---------- ---------- ---------- ---------- ---------- ----------
A25   -------------------- ---------- ---------- ---------- ---------- ---------- ---------- ---------- ---------- ----------
A13   -------------------- ---------- ---------- ---------- ---------- ---------- ---------- ---------- ---------- ----------
A18   -------------------- ---------- ---------- ---------- ---------- ---------- ---------- ---------- ---------- ----------
A27
A3    -------------------- ---------- ---------- ---------- ---------- ---------- ---------- -------/// ////////// //////////
A21   -------------------- ---------- ---------- ---------- ---------- ---------- ---------- -------/// ////////// //////////
A12   -------------------- ---------- ---------- ---------- ---------- ---------- ---------- ---------- ---------- ----------
A20   ---agc-------------- ---------- ---------- ---------- ---------- ---------- ---------- ---------- ---------- ----------
A26   -------------------- ---------- ---------- ---------- ---------- ---------- ---------- ------c--- ---------- g--------Δ
A4    ---ΔΔΔΔ----------ggΔ ---------- --+------- ---------- -ΔΔΔΔ----- ---------- a--------- g-tga-c--- ----g-g--- g---------
A23   ---+-------g------g- ---------- --+------- ---------- ------//// //////---- ---------- ---------- ---------- ----------

         330        340        350        360        370        380        390        400        410        420        430
Sμ    GCTGGGTGAC CTGAGCTGAG CTGAGCTGGG TGAGCTGAGC TGGGGTGAGC TGAGCTGAGC TGGGGTGAGC TGAGCTGAGC TGAGCTGGGT GAGCTGAGCT GGGGTGAGCT
A1    -------------------- ---------- ---------- ---------- ---------- ---------- ---------- ---------- ---------- ----------
A30   -------------------- ---------- ---------- ---------- ---------- ---------- ---------- ---------- ---------- ----------
A10   -------------------- ---------- ---------- ---------- ---------- ---------- ---------- ---------- ---------- ----------
A2    -------------------- ---------- ---------- ---------- ---------- ---------- ---------- ---------- ---------- ----------
A25   -------------------- ---------- ---------- ---------- ---------- ---------- ---------- ---------- ---------- ----------
A13   -------------------- ---------- ---------- ---------- ---------- ---------- ---------- ---------- ---------- ----------
A18   -------------------- ---------- ---------- ---------- ---------- ---------- ---------- ---------- ---------- ----------
A27
A3    //////////////////// ////////// ////////// ////------ ---------- ---------- ---------- ---------- ---------- ----------
A21   //////////////////// ////////// ////////// ////------ ---------- ---------- ---------- ---------- ---------- ----------
A12   ---ΔΔΔΔΔΔΔΔΔΔΔΔΔΔΔΔΔ ΔΔΔΔΔΔΔΔΔΔ ΔΔΔΔΔΔΔΔΔΔ ΔΔΔΔΔΔΔΔΔΔ ΔΔΔΔΔΔΔΔΔΔ ΔΔ-------- ---------- ---------- ---------- ----------
A20   -------------------- ---------- ---------- ---------- ---------- ---------- ---------- ---------- ---------- ----------
A26   ΔΔΔ---------g------- ---------- ---------- ---------- ---------- ---------- ---------- ---------- ---------- ----------
A4    ----+--------------- ---g-g--a- ---------- ---------- ---------- ---------- ------ag-g ---a-----c tg-g------ --a-------
A23   -------------------- ---------- ---------- ----c----- -------g-Δ --a-c----- ---a------ ---------- ****

         440        450        460        470        480        490        500        510        520        530        540
Sμ    GAGCTGAGCT GGGGTGAGCT GAGCTGAGCT GGGGTGAGCT GAGCTGAGCT GGGGTGAGCT GAGCTGAGCT GGGGTGAGCT GAGCTAGGGT GAACTGGGCT GGGTGAGCTG
A1    ---------- ---------- ---------- ---------- ---------- ---------- ---------- ---------- ---------- ---------- ----------
A30   ---------- ---------- ---------- ---------- ---------- ---------- ---------- ---------- ---------- ---------- ----------
A10   ---------- ---------- ---------- ---------- ---------- ---------- ---------- ---------- ---------- ---------- ----------
A2    ---------- ---------- ---------- ---------- ---------- ---------- ---------- ---------- ---------- ---------- ----------
A25   ---------- ---------- ---------- ---------- ---------- ---------- ---------- ---------- ---------- ---------- ----------
A13   ---------- ---------- ---------- ---------- ---------- ---------- ---------- ---------- ---------- ---------- ----------
A18   ---------- -----****
A27
A3    ---------- ---------- ---------- ---------- ---------- ---------- ---------- ------****
A21   ---------- ---------- ---------- ---------- ---------- ---------- ---------- ---------- ---------- ---------- ----------
A12   ---------- ---------- ---------- ---------- ---------- ---------- ---------- ---------- ---------- ---------- ----------
A20   ---------- ---------- ---------- ---------- ---------- ---------- ---------- ---------- ---------- ---------- ----------
A26   ---------- ---------- ---------- ----****
A4    --------ΔΔ ΔΔΔ----a-- ****
A23

         550        560        570        580        590        600        610        620        630        640        650
Sμ    GAGTGAGCTG AGCTGAGGTG AACTGGGGTG AGCCGGGATG TTTTGAGTTG AGCTGGGGTA AGATGAGCTG AACTGGGGTA AGATGGGATG AGCTGTGGTG AGGGGAGCTG
A1    ---------- ---------- ---------- ---------- ---------- ---------- ---------- ---------- ---------- ---------- ----------
A30   ---------- ---------- ---------- ---------- ---------- ---------- ---------- ---------- ---------- ---------- ----------
A10   ---------- ---------- ---------- ---------- ---------- ---------- ---------- ---------- ---------- ---------- ----------
A2    ---------- ---------- ---------- ---------- ---------- ---------- ---------- ---------- ---------- ---------- ----------
A25   ---------- ---------- ---------- -------****
A13   ---------- ---------- ---------- ---------- ---------- --------****
A18
A27
A3
A21   ---------- ---------- ---------- ----////// ////////// ////////// ////////// /////////g --cc------ --------****
A12   ---------- ---------- ---------- ---------- ---------- ---------- ---------- ---------- ---------- ---------- ----****
A20   ---------- ---------- ---------- ---------- ---------- ---------- ---------- ---------- ---------- ---------- ----****
A26
A4
A23

         660        670        680        690        700        710        720        730        740        750        760
Sμ    GATTGAACTG AGCTGTGTGA GCTGAGCTGG GGTCAGCTGA GCAAGAGTGA GTAGAGCTGG CTGGCCAAGA ACCAAGAATC AATTAGGCTA AGTGAGCCAG ATTGCGCTGG
A1    ---------- ---------- ---------- ---------- ---------- --****
A30   ---------- ---------- ---------- ---------- ---------- ****
A10   ---------- ---------- ---------- -****
A2    ---------- ---------- ---------- -****
A25
A13
A18
A27
A3
A21
A12
A20
A26
A4
A23
```
