# Supplementary figures and images for: Topoisomerase I Plays a Critical Role in Suppressing Genome Instability at a Highly Transcribed G-Quadruplex-Forming Sequence
Source: PLoS Genet. 2014 Dec 4;10(12):e1004839. doi: 10.1371/journal.pgen.1004839 (PMC4256205; doi:10.1371/journal.pgen.1004839)

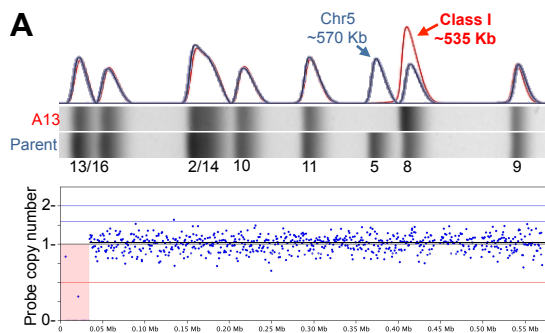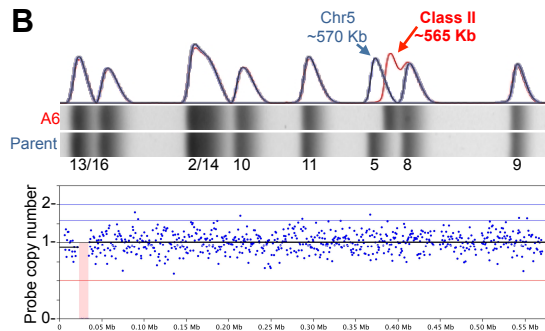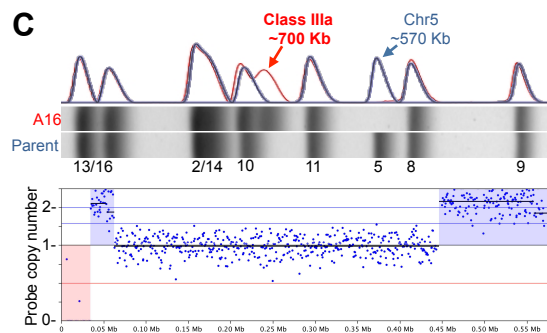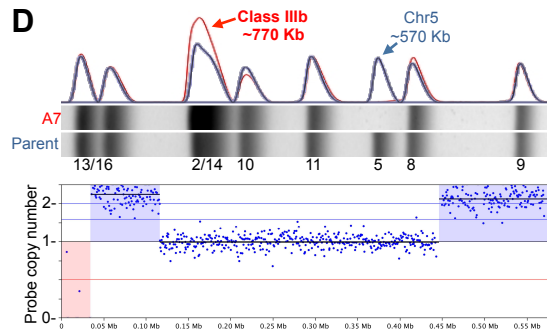

Supplement: Figure S2 — Molecular karyotype analysis of CHR5 GCRs. A. GCR class I, clone A13; B. GCR class II, clone A6; C. GCR class IIIa, clone A16; and D. GCR class IIIb, clone A7. In all panels, the top part corresponds to sections of the same PFGE gel shown in Fig. 2A, cropped between the CHR13/6 and CHR9 regions. Directly above the PFGE images are the superimposed plots of the quantitative trace analysis of the PFGE lanes for the parental strain (dark blue trace) and for the respective GCR clone (red trace). The image pixel intensity traces (vertical axis) for the GCR PFGEs were normalized relative to the trace of the parental strain. The parental and GCR traces closely overlapped for all chromosomes other than CHR5 and the rearranged CHR5 in the various GCR classes. The approximate size of CHR5 and the GCRs are indicated. The lower part of each panel shows the array-CGH copy number plots for the probes (blue dots) from CHR5 in the respective GCR clones. The vertical axis corresponds to the Log2(GCR[Cy5]/Parent[Cy3]) signal and the corresponding positions for 0, 1, and 2 copies of genomic material in the respective GCR clones. The horizontal axis corresponds to the physical position of each probe on CHR5. Regions where full deletions where detected are shaded in red, and regions where duplications were detected are shaded in light blue. The specific breakpoints in each GCR class are summarized in main text and in Fig. 2B. (PDF) [file pgen.1004839.s002.pdf]

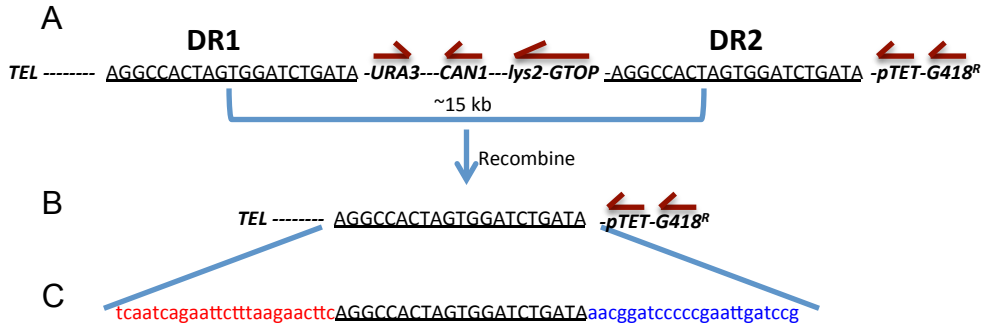

Supplement: Figure S3 — The direct repeat-mediated deletions in Class II GCR events. A. The locations of the 21 nt direct repeats (underlined) before the deletion are shown relative to the relevant genetic features. Directions of transcription for genes listed are shown with red arrows. DR1 originated from pUG72 plasmid used to generate the loxP-URA3Kl-loxP cassette, which replaced the HXT13 ORF. DR2 originated from pCM225 plasmid used to replace pLYS2 with pTET. B. The genomic configuration after the 15 kb deletion between the 21 nt direct repeats. C. The sequences flanking the 21 nt sequence after the 15 kb deletion (in lower case letters) were confirmed by PCR-sequencing to be from HXT13-proximal (in red) and pTET-proximal (in blue). (PDF) [file pgen.1004839.s003.pdf]

**A****Lys<sup>-</sup>**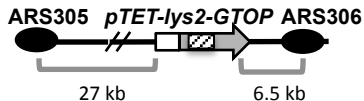

↓ Gene  
Conversion

**Lys<sup>+</sup>**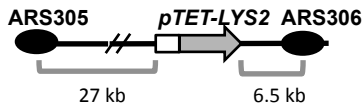**B**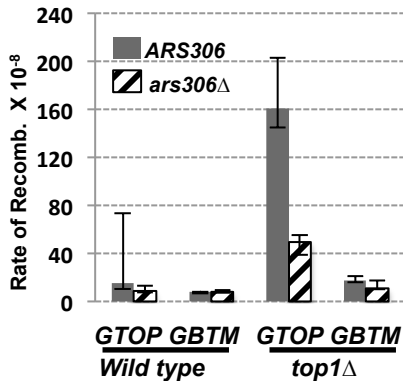

Supplement: Figure S6 — LOH classes not initiated at the pTET-lys2-GTOP or -GBTM cassette. Schematic representations of mitotic recombination products resulting in the selected the 5-FOAR LOH events are shown. LOH classe E initiated at the pTET-lys2-GTOP or -GBTM cassette are shown in Figure 3A. YPH45-CHR3 and YJM789-CHR3 are represented by black and red lines, respectively. Location of the pTET-lys2-GTOP or –GBTM cassette on the left arm of CHR3 is indicated by the hashed box. Green boxes indicate the approximate locations of the heterozygous SNP markers listed in Table S2. Telomeres are represented as “tgtgtg”. Hemizygous URA3 and G418R markers present only on the YPH45-CHR3 homolog are indicated by blue boxes. LOH classes A–G are defined by RFLP-SNP assay. Briefly, SNPs between the YJM789 and YPH45 CHR3 sequences were identified by comparing the genome sequences (Saccharomyces Genome Database (SGD). Then, five sites were chosen where the SNP either generates or abolishes a restriction enzyme recognition site. At a location equivalent to the SGD coordinate 54440 in S288c, for example, a StyI cut site (CCAAGG) is present on YPH45-derived CHR3. On YJM789 CHR3, this sequence differs by one nucleotide (CCAAGA), and cannot be cut by StyI. In a diploid carrying both copies of CHR3 with this region unchanged (heterozygous), PCR amplification followed by StyI digestion will detect both cut (YPH45) and uncut (YJM789) alleles. If a recombination event in the heterozygous diploid results in the loss of this region of YPH45-CHR3, only the uncut YJM789 fragment will be present. Additionally, we tested by PCR whether the hemizygous URA3 and G418R markers, were retained in the 5-FOAR isolates. All sites surveyed with the RFLP-SNP assay are listed in Table S2. (PDF) [file pgen.1004839.s006.pdf]

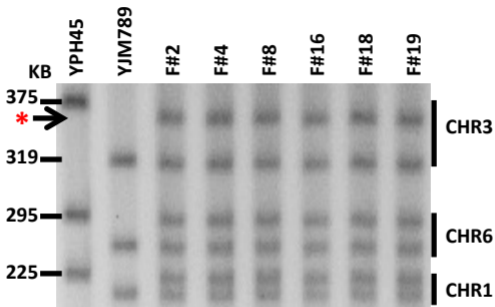

Supplement: Figure S7 — PFGE analysis of LOH (5-FOAR) isolates. YPH45/YJM789 hybrid CHR3 with size estimated at 362 kb is indicated with the red asterisk. Parental haploids YPH45 and YJM789 are each indicated. Lanes F#2–F#19 are 5-FOAR diploid isolates derived from the strain containing pTET-lys2-GTOP (OPPO) cassette in top1Δ background. (PDF) [file pgen.1004839.s007.pdf]
