# Supplementary material for: Topoisomerase I Plays a Critical Role in Suppressing Genome Instability at a Highly Transcribed G-Quadruplex-Forming Sequence
Source: PLoS Genet. 2014 Dec 4;10(12):e1004839. doi: 10.1371/journal.pgen.1004839 (PMC4256205; doi:10.1371/journal.pgen.1004839)
Supplement: Figure S4 — Fold-back inverted duplications originating at the G-quadruplex sequence. A and B. The DNA sequence determined from the inverted duplication structures in clone A7 (A) and A8 (B) are shown at the bottom of each figure (Step 5), and the proposed mechanism of formation is shown above them. Step 1. A DNA double strand break forms within or proximal to the Sμ sequence. Step 2. 5′ to 3′ resection creates a ssDNA region. Step 3. The 3′ end of the ssDNA folds back on itself forming a loop of 59 nt (A – clone A7) or 441 nt (B – clone A8) (nucleotides in green) and anneals to a 4 nt microhomology (underlined). Step 4. The annealed 3′ end primes break-induced replication (nucleotides in blue) going toward the right arm. Step 5. After BIR, a complementary DNA strand is synthesized (nucleotides in red) to complete the inverted chromosomal rearrangement event. Regions deleted and duplicated in the resulting clone are shaded in red and blue, respectively. The arrows above and below the DNA sequence show the two sides of the inverted duplication, which flank the microhomologies and the single copy region corresponding to the original ssDNA loop. C. Models for the generation of the recovered Class III GCRs. The inverted duplication rearrangements initiated by a double strand break lesion at the Sμ sequence. Unique regions in CHR5 are labeled with letters A through F. The CHR5 fragment to the left of Sμ containing TEL05L, URA3, and CAN1 is lost (segment A), while the resected 3′ end of the CHR5 fragment to the right of Sμ folds back on itself and initiates BIR at the B segment. At this point, two possible models are shown to explain the structure of the recovered clones. Model 1: Template switching. 1A. BIR proceeds from left to right through the B and C segments (green line) until reaching a dispersed Ty or LTR repeat (open white arrow). 1B. At this point the BIR fork collapses, and re-anneals at a homologous Ty or LTR on the right arm, then resumes extension copying the F segment [file pgen.1004839.s004.pdf]

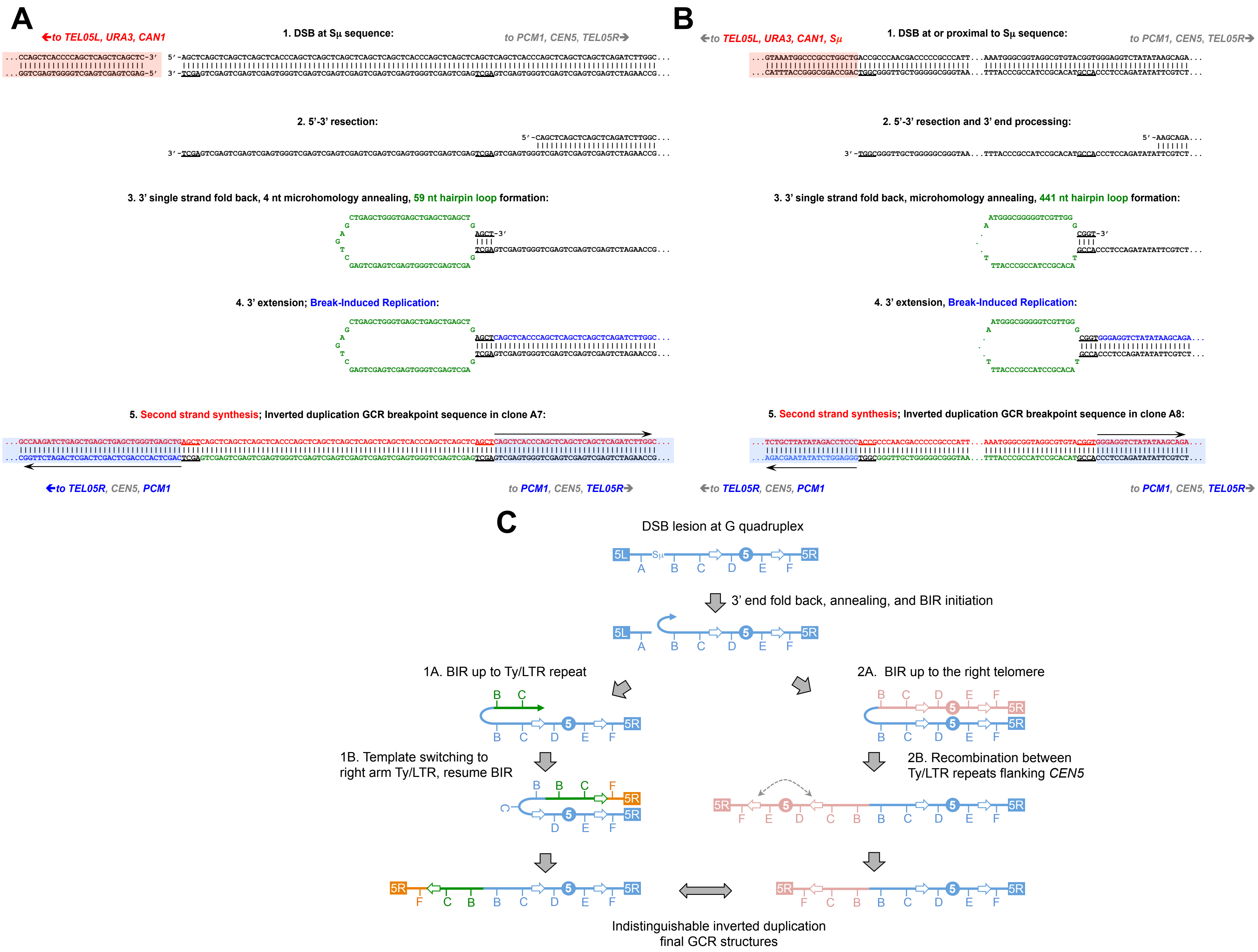
A
to TEL05L, URA3, CAN1
1. DSB at Sμ sequence:
to PCM1, CEN5, TEL05R
2. 5'-3' resection:
3. 3' single strand fold back, 4 nt microhomology annealing, 59 nt hairpin loop formation:
4. 3' extension; Break-Induced Replication:
5. Second strand synthesis; Inverted duplication GCR breakpoint sequence in clone A7:
to TEL05R, CEN5, PCM1
to PCM1, CEN5, TEL05R
B
to TEL05L, URA3, CAN1, Sμ
1. DSB at or proximal to Sμ sequence:
to PCM1, CEN5, TEL05R
2. 5'-3' resection and 3' end processing:
3. 3' single strand fold back, microhomology annealing, 441 nt hairpin loop formation:
4. 3' extension, Break-Induced Replication:
5. Second strand synthesis; Inverted duplication GCR breakpoint sequence in clone A8:
to TEL05R, CEN5, PCM1
to PCM1, CEN5, TEL05R
C
DSB lesion at G quadruplex
5L
Sμ
5
5R
A
B
C
D
E
F
3' end fold back, annealing, and BIR initiation
1A. BIR up to Ty/LTR repeat
2A. BIR up to the right telomere
1B. Template switching to right arm Ty/LTR, resume BIR
2B. Recombination between Ty/LTR repeats flanking CEN5
Indistinguishable inverted duplication final GCR structures
