# Supplementary material for: Topoisomerase I Plays a Critical Role in Suppressing Genome Instability at a Highly Transcribed G-Quadruplex-Forming Sequence
Source: PLoS Genet. 2014 Dec 4;10(12):e1004839. doi: 10.1371/journal.pgen.1004839 (PMC4256205; doi:10.1371/journal.pgen.1004839)
Supplement: Figure S5 — Reciprocal crossover (RCO) and break induced replication (BIR) producing 5-FOAR recombinants. A simplified diagram depicting RCO or BIR between the heterozygous CHR3s in LOH assay (see Fig. 3). YPH45-derived or YJM789-derived CHR3s are depicted in black or red lines, respectively. Centromeres are represented with gray circle in each diagram. The diploid cell in S-phase with duplicated CHR3s is shown at the top. After repairing DNA breaks by RCO or BIR, two progenies (one 5-FOAR and one 5-FOAS) produced after chromosome segregation and cell division are depicted at the bottom. BIR and RCO will produce one progeny containing one copy or two copies of URA3 (shaded in gray), respectively, which will not grow on the 5-FOA-containing selection media. The 5-FOAR progenies from RCO and BIR (not shaded) are identical. Because we are unable to analyze the 5-FOAS product of the mitosis, we cannot distinguish whether the recombinant CHR3s observed are products of RCO or BIR, which produce identical 5-FOAR daughters. (PDF) [file pgen.1004839.s005.pdf]

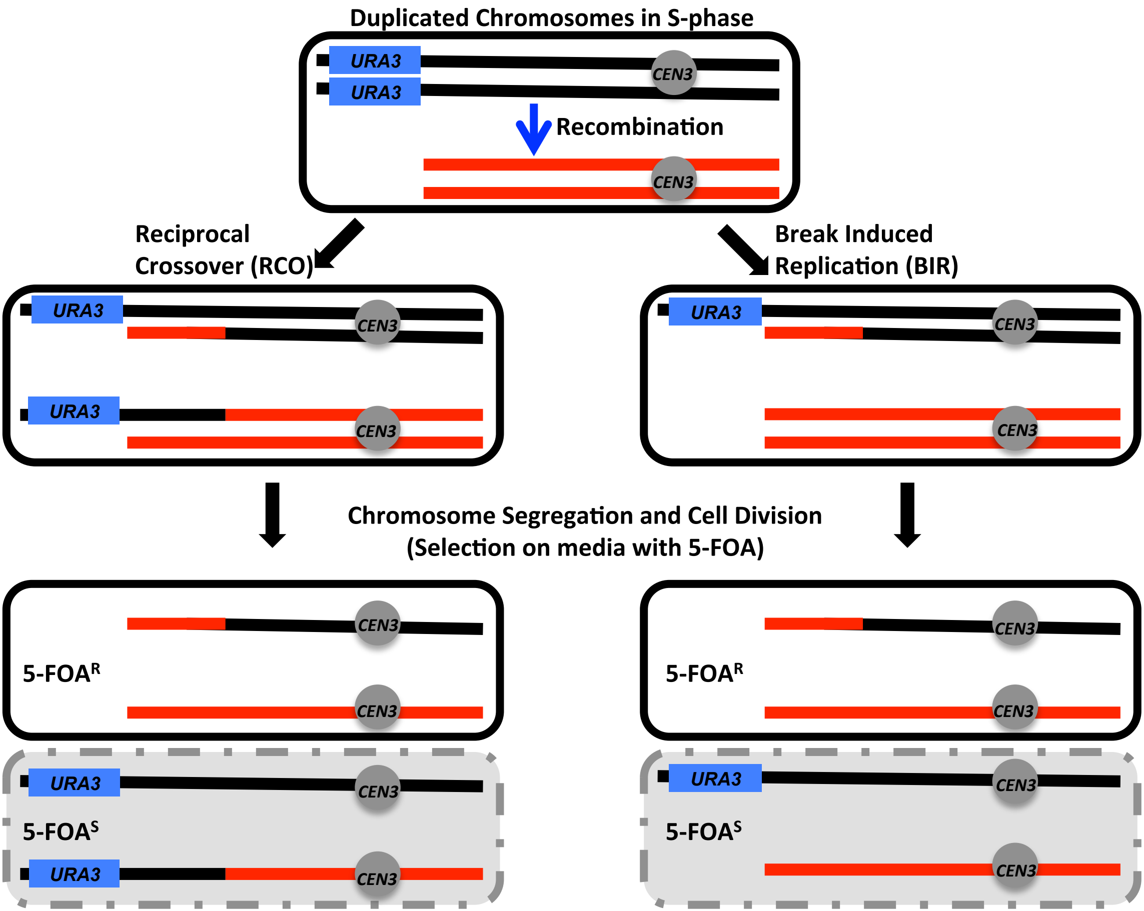
Duplicated Chromosomes in S-phase
URA3
URA3
CEN3
Recombination
CEN3
Reciprocal Crossover (RCO)
Break Induced Replication (BIR)
URA3
CEN3
URA3
CEN3
URA3
CEN3
CEN3
Chromosome Segregation and Cell Division
(Selection on media with 5-FOA)
CEN3
5-FOA^R
CEN3
CEN3
5-FOA^R
CEN3
URA3
CEN3
5-FOA^S
URA3
CEN3
URA3
CEN3
5-FOA^S
CEN3
