# Supplementary material for: Topoisomerase I Plays a Critical Role in Suppressing Genome Instability at a Highly Transcribed G-Quadruplex-Forming Sequence
Source: PLoS Genet. 2014 Dec 4;10(12):e1004839. doi: 10.1371/journal.pgen.1004839 (PMC4256205; doi:10.1371/journal.pgen.1004839)
Supplement: Table S1 — The relative RNA levels of lys2-GTOP or -GBTM allele in top1Δ backgrounds. (PDF) [file pgen.1004839.s008.pdf]

Table S1. The relative RNA levels of *lys2-GTOP* or *-GBTM* allele in *top1*Δ backgrounds.

lys2 allele	*Doxycycline*	*Relative RNA - 5' end* (% pTET-lys2-GTOP; no doxycycline)*	*Relative RNA - 3' end** (% pTET-lys2-GTOP; no doxycycline)*
pTET-lys2-GTOP	None	100 (±17)***	100 (±33)***
pTET-lys2-GTOP	2 μg/ml	0.45 (±0.06)***	0.59 (±0.27)***
pTET-lys2-GBTM	None	155 (±7.0)***	54 (±12)***
pTET-lys2-GBTM	2 μg/ml	1.04 (±0.25)***	0.82 (±0.12)***

RNA was extracted using the standard hot acidic phenol method and treated with DNase I (New England Biolabs). Relative RNA levels were determined by quantitiative RT-PCR and ΔΔCq analysis using *ACT1* as the reference gene. RT-PCR was carried out using SensiFAST SYBR No-ROX One-Step kit from Bioline and Biorad CFX machine. Primers for amplification of 5' end (*) are located ~ 250 NT from the 5' end of the *LYS2* mRNA and ~ 100 NT upstream of *GTOP* or *GBTM* cassette-insertion site. Primers for the amplification of 3' end (**) are located ~4,000 NT from the 5' end of the *LYS2* mRNA. Primer sequences will be available upon request. The standard deviations (***) were calculated from three independent experiments.
