# Supplementary material for: Topoisomerase I Plays a Critical Role in Suppressing Genome Instability at a Highly Transcribed G-Quadruplex-Forming Sequence
Source: PLoS Genet. 2014 Dec 4;10(12):e1004839. doi: 10.1371/journal.pgen.1004839 (PMC4256205; doi:10.1371/journal.pgen.1004839)
Supplement: Table S2 — SNPs or hemizygous markers used for RFLP-SNP assay. (PDF) [file pgen.1004839.s009.pdf]

Table S2. SNPs or hemizygous markers used for RFLP-SNP assay.

*SGD coordinate**	*Marker tested /Enzyme for cutting*	*In YPH45*	*In YJM789*
22000	*URA3*	Present	Absent
54430	*Sty*I	TCCAAGGA	TCCAAGAA
59250	*BsrB*I	GACGCTCT	GCCGCTCT
62240	*Spe*I	GACTAGTG	GACTAATG
68300	$G418^{R}$	Present	Absent
78380	*Nar*I	TGGTGCCA	TGGCGCCA
152550	*Hind*III	AAAACTTA	AAAGCTTA

*Coordinates are approximate and correspond to S288c reference sequence.
For *URA3* or $G418^{R}$, presence or absence of the PCR band was tested. For all other markers, PCR products were digested with the indicated enzymes; cutting sites are shown underlined.
